# Using carpet plots to analyze transit times of low frequency oscillations in resting state fMRI

**DOI:** 10.1038/s41598-021-86402-z

**Published:** 2021-03-26

**Authors:** Bradley Fitzgerald, Jinxia Fiona Yao, Thomas M. Talavage, Lia M. Hocke, Blaise deB Frederick, Yunjie Tong

**Affiliations:** 1 Weldon School of Biomedical Engineering, Purdue University, 206 S. Martin Jischke Drive, West Lafayette, IN 47907-2032 USA; 2 School of Electrical and Computer Engineering, Purdue University, West Lafayette, IN 47907 USA; 3 Mclean Hospital, Harvard Medical School, Belmont, MA 02478 USA

**Keywords:** Neuroscience, Blood flow

## Abstract

A “carpet plot” is a 2-dimensional plot (time vs. voxel) of scaled fMRI voxel intensity values. Low frequency oscillations (LFOs) can be successfully identified from BOLD fMRI and used to study characteristics of neuronal and physiological activity. Here, we evaluate the use of carpet plots paired with a developed slope-detection algorithm as a means to study LFOs in resting state fMRI (rs-fMRI) data with the help of dynamic susceptibility contrast (DSC) MRI data. Carpet plots were constructed by ordering voxels according to signal delay time for each voxel. The slope-detection algorithm was used to identify and calculate propagation times, or “transit times”, of tilted vertical edges across which a sudden signal change was observed. We aim to show that this metric has applications in understanding LFOs in fMRI data, possibly reflecting changes in blood flow speed during the scan, and for evaluating alternative blood-tracking contrast agents such as inhaled CO_2_. We demonstrate that the propagations of LFOs can be visualized and automatically identified in a carpet plot as tilted lines of sudden intensity change. Resting state carpet plots produce edges with transit times similar to those of DSC carpet plots. Additionally, resting state carpet plots indicate that edge transit times vary at different time points during the scan.

## Introduction

Resting state functional magnetic resonance imaging (rs-fMRI) records spontaneous fluctuations of the blood-oxygen-level-dependent (BOLD) signal from individuals lying quietly in the scanner without performing any specific task^1^. It has become a powerful, widely adopted tool to study functional organization^2^ and brain networks^3^, as well as their changes related to health and disease^4^.

BOLD fMRI does not measure neuronal signals directly. It is a blood-related signal, which reflects a dynamic interplay between cerebral blood flow (CBF), volume, and cerebral metabolic rate of oxygen consumption^5^. Thus, it measures neuronal activation indirectly through neurovascular coupling^6^. As a result, it is sensitive to physiological fluctuations (non-neuronal), such as those arising from respiratory and cardiac cycles.

One of the physiological fluctuations to which BOLD fMRI is sensitive is systemic low frequency oscillations (sLFOs). sLFOs are a subset of low frequency oscillations (LFOs; 0.01 ~ 0.1 Hz) that are widespread in BOLD fMRI signals from the brain and form a major component of the global fMRI signal. LFOs, as general signals, are comprised of signals from both neuronal and general physiological origins. sLFOs represent the portion of LFOs that (1) has a physiological origin, and (2) propagates through the brain. sLFOs with minimal neuronal influence are commonly obtained from resting state BOLD signals in large blood vessels (e.g. superior sagittal sinus, internal carotid artery) or from global means to minimize neuronal contamination. Previous rs-fMRI studies have found sLFOs in different voxels at different time delays^7–9^. Recently, sLFOs have even been used as an endogenous contrast agent to track blood flow in the brain in rs-fMRI data^10–12^.

While the dynamic pattern of sLFOs moving through the brain is similar to that of blood in both sequence and timing, questions remain regarding sLFOs and their relation to blood flow. First, how accurate is a hemodynamic parameter (such as transit time) calculated using sLFOs compared to that calculated using dynamic susceptibility contrast (DSC) imaging? DSC imaging can serve as a reliable MRI reference standard for measuring blood flow in the brain, using the injection of a gadolinium contrast agent and imaging its passage through the brain^13^. Second, the origins and characteristics of sLFOs are still not entirely clear; sLFOs have been found to be associated with vasomotion^14^, Mayer waves^15^, respiration depth and heart rate changes^5,16–18^. A detailed discussion of the potential sources of the sLFOs can be found in a separate review paper^19^. However, recent studies on humans and animals have found that some portion of the pervasive low frequency physiological signal (i.e., global fMRI signal) is neuronal and closely related to alertness, which is supported by concurrent electrocorticography measurements^20^. It is critical to answer these questions in order to better utilize sLFOs and help improve rs-fMRI network analyses.

The carpet plot, which takes the form of a heatmap showing signal intensity, is a 2-D voxel vs. time matrix which has been adopted in many studies^21–23^ and incorporated in analytical pipelines^23^ to assess the general quality of fMRI data. Previously, the main application of the carpet plot has been to detect/visualize motion artifacts, which generally affect the whole brain at one particular time. Power et al. have also identified widespread vertical broad bands of rs-fMRI in carpet plots^22^, called widespread signal deflections, which represent the (global) LFOs in the BOLD signals.

**Figure 1:**
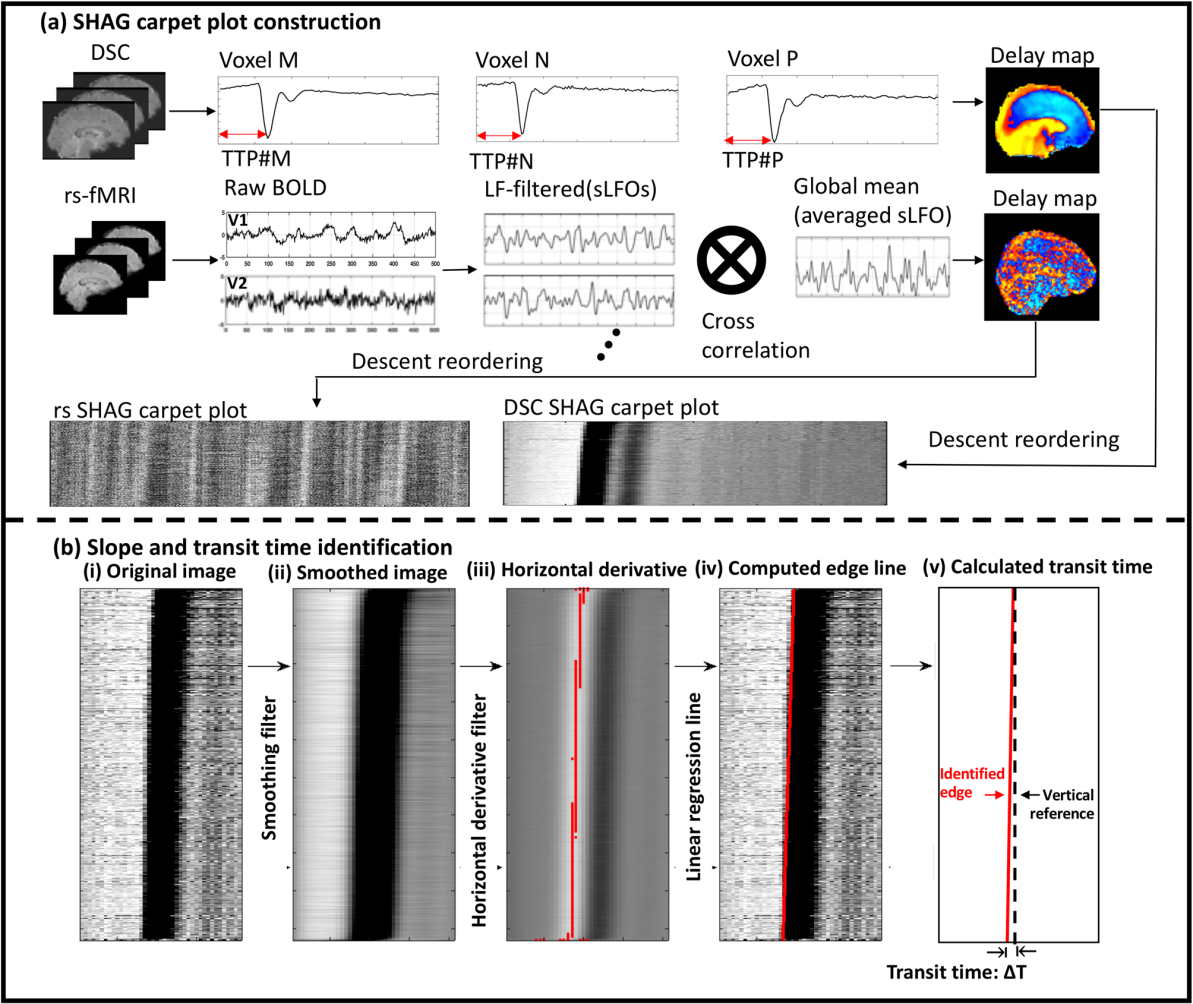
Demonstration of carpet plot reconstruction and slope identification. (**a**) Carpet plot reconstruction for DSC-MRI and for rs-fMRI. Carpet plots for DSC-MRI scans were constructed by reordering voxels in descending order based on time to peak (TTP). Carpet plots for rs-fMRI were reconstructed by reordering voxels based on the delay time derived from the cross-correlation between the sLFO signal for a given voxel and the averaged global sLFO across all voxels in the brain. MRI data processing and image visualization was assisted by use of the FMRIB Software Library (FSL, v5.0 for DSC and rs-fMRI data, v6.0 for the CO_2_-MRI data, https://fsl.fmrib.ox.ac.uk/fsl/fslwiki)^24^. (**b**) Slope-detecting algorithm developed for computation of carpet plot edge slopes and transit times. The original image (i) is smoothed with a 2D blurring filter (ii), followed by a horizontal derivative filter (iii). For every row of (iii), the maximum derivative location is located and denoted by a red point. These points are used as data to compute a linear regression line or edge, shown in (iv), from which the slope (steepness of the edge) and the transit time (time difference between the bottom and top of an identified edge indicating the time taken for the corresponding fluctuation to traverse all voxels in the carpet plot) can be calculated.

In this study, we propose and evaluate a novel application of carpet plots in which voxels are reordered according to the “arrival time” of the sLFO within each voxel. We call these ordered carpet plots Sorted Hemodynamic Arrival Graphs, or SHAG carpet plots. We utilize these SHAG carpet plots to study the propagation time of sLFOs in rs-fMRI, leading to our ability (1) to compare hemodynamic measurements made using sLFOs with those derived from DSC-MRI; (2) explore the potential of assessing blood flow speed moment by moment during the rs-fMRI scan; and (3) characterize the propagation speed of predicted neuronal and physiological (vascular) components of sLFOs (or global means). This is done through the application of an in-house slope-detection algorithm used to detect and quantify edges in carpet plots present due to sLFOs in the data. Calculated edge slopes are used to compute an edge’s “transit time”, or the horizontal (i.e. time axis) distance traversed by the edge (see Fig. 1b). These edges represent the propagation of some signal pattern throughout the majority of voxels in the brain.

One of the overarching goals of this method is to help provide an understanding of sLFOs in rs-fMRI data, a phenomenon which is not yet fully explained. We believe this methodology to be beneficial because it produces a simple metric, transit time, which can be used to compare propagation speeds of sLFOs or a blood-based bolus within and across subjects. This metric can be used to help understand how the propagation of sLFOs relate to the propagation of purely blood-based propagation phenomena, like that observed in DSC-MRI. Further, the transit time metric can be used to evaluate how sLFO propagation speeds vary over time in a subject, creating the potential to temporally link changes in sLFO propagation with events such as neuronal activity (e.g., autonomic nervous system), which can shed light on the causes of sLFOs. A second overarching goal (to be explored in a future study) is to apply the use of the transit time metric to evaluate alternative blood-tracking agents such as inhaled CO_2_—which is non-invasive compared to DSC-MRI and offers higher signal-to-noise ratio (SNR) compared to rs-fMRI—against standards such as DSC-MRI.

To these ends, we first apply SHAG carpet plots to DSC data to validate our method. For DSC-MRI, signal intensity changes dramatically with the arrival of the injected bolus in the bloodstream, meaning that edge transit times can provide information on blood flow speed with high temporal accuracy (within the limits of the temporal resolution provided by the imaging sequence). Edges with a higher slope (meaning a shorter transit time) represent faster blood flow. Then, we apply the method to rs-fMRI. For rs-fMRI, edge transit times reflect a potential interplay between the speed of blood flow and propagation speed of neuronal signal oscillations. Finally, we briefly explore the viability of CO_2_ inhalation as an alternative blood-tracking bolus through the analysis of transit times from a SHAG carpet plot computed from CO_2_ challenge MRI data.

## Results

DSC-MRI scans (using a gadolinium contrast injection) and rs-fMRI scans of eight healthy subjects (1F, 7 M, mean ± s.d., 33 ± 12 years), all acquired on a 3 T Siemens MRI scanner, were used in this study (this study represents a reanalysis of DSC data from a previous study conducted by Tong et al.^10^). Both DSC-MRI and rs-fMRI SHAG carpet plots were generated by reordering the timeseries of voxels based on their relative signal delays, though these delays were determined independently for DSC and rs-fMRI data (see Fig. 1a). The voxel-specific delay for DSC-MRI data was represented by time to peak (TTP), which is defined as the time duration from the beginning of the scan to the dip of the DSC signal loss. The voxel-specific delay for rs-fMRI was represented by the relative delay between the sLFOs of BOLD signals from the global mean signal (as a reference) to any given voxel (see Methods). For both DSC and rs-fMRI data, SHAG carpet plots were constructed by vertically concatenating the scaled, demeaned, unfiltered BOLD fMRI time series for all voxels based on their corresponding delay times in a descending order**.** Both DSC-MRI and rs-fMRI SHAG carpet plots were cropped by removing noise voxels, for which there is no clear consistent pattern recognized by visual inspection (see Methods). The time difference between the bottom and top of an identified edge indicates the time taken for the corresponding fluctuation to traverse all voxels in the carpet plot, referred to here as “transit time” (see Fig. 1b, v).

### Characteristics of the SHAG carpet plots

**Figure 2:**
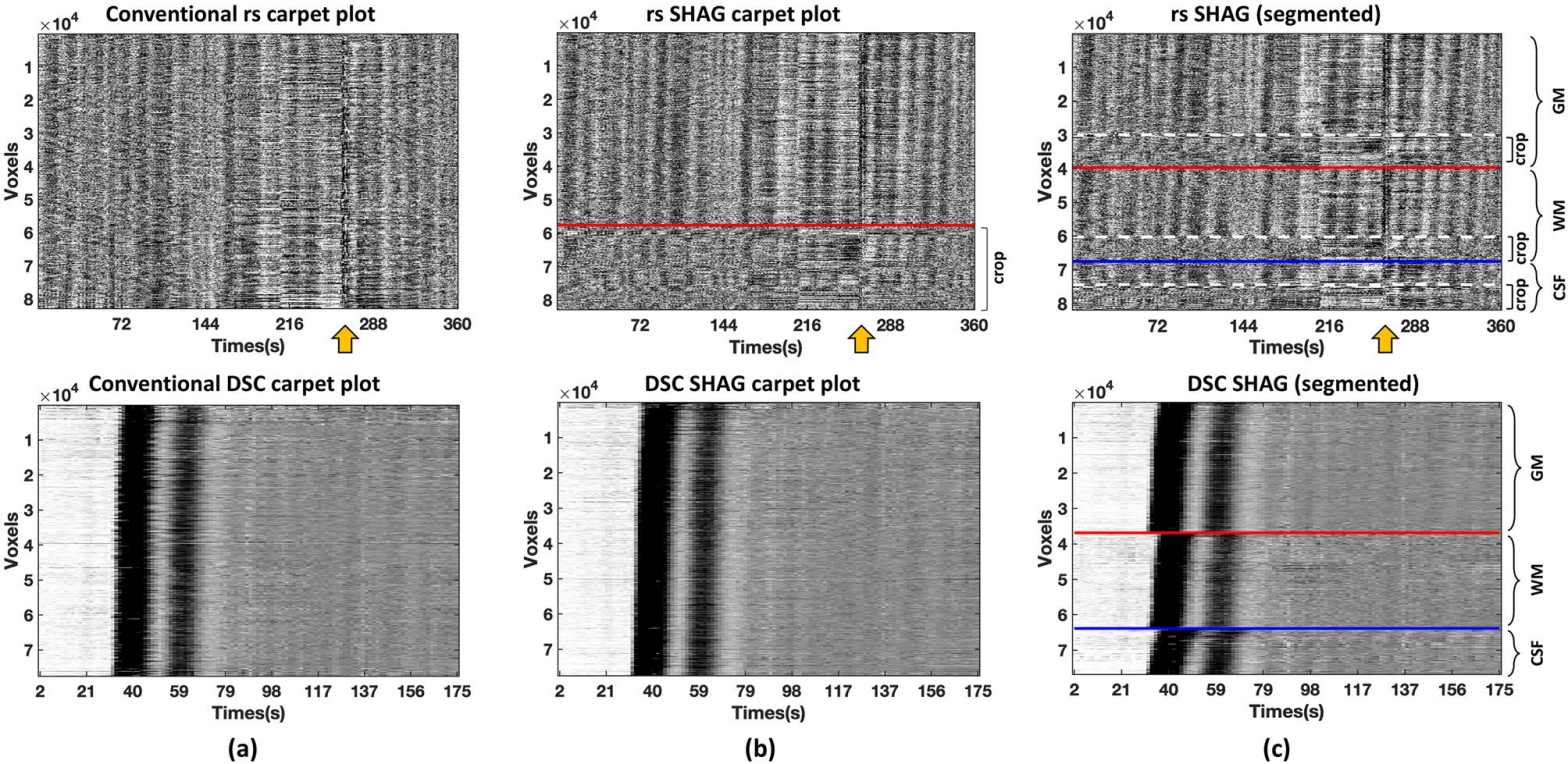
Comparison of conventional carpet plot and SHAG carpet plot for rs-fMRI (top row) and DSC-MRI (bottom row). Column (**a**) presents conventional carpet plots with no voxel ordering. Column (**b**) presents SHAG carpet plots with reordered voxels. For the rs SHAG carpet plot in the top row, voxels below the red line are those which would later be removed (cropped) from the carpet plot in order to perform edge detection. We note that voxels were similarly cropped from DSC SHAG carpet plots, however the percentage of voxels was so small that it could not be clearly labeled in this figure. Column (**c**) presents SHAG carpet plots segmented by the predominant tissue type in the voxel (gray matter (GM), white matter (WM), and cerebrospinal fluid (CSF)). GM, WM, and CSF masks were generated by MATLAB-based software (v1.0, http://doi.org/10.5281/zenodo.3871362)^25^. For the segmented rs SHAG carpet plot, the white dotted line in each tissue section separates the voxels which would later be cropped. Yellow arrows indicate a motion artifact.

DSC and rs-fMRI carpet plots are displayed in the lower and upper rows of Fig. 2, respectively. Conventional, unordered carpet plots are shown in column (a) and the SHAG carpet plots (our novel method) are shown in column (b). These carpet plots reveal widespread signal fluctuations (i.e., consistent signal intensity changes in the majority of the voxels). However, the DSC and rs-fMRI carpet plots each have unique reasons for these patterns. For DSC, the prominent fluctuation is caused by the T2* signal drop induced by the arrival of the gadolinium bolus. For rs-fMRI, it is caused by the alignment of peaks and troughs of the widespread sLFOs existing in rs-fMRI signals for the majority of the voxels. Edges of sudden intensity change are much more tilted in the SHAG carpet plots (column (b)) than in conventional, unordered carpet plots (column (a)).

Voxels that do not have clear and consistent patterns are grouped at the bottom of each reordered carpet plot (see Fig. 2, column (b), 0.8 ± 0.7 (s.d.) % of all voxels for DSC and 28.7 ± 5.2 (s.d.) % for rs-fMRI). These voxels were not concentrated in any particular brain region but appeared in regions of white matter (WM), gray matter (GM), and cerebrospinal fluid (CSF). This is illustrated in Fig. 2, column (c) as a segmented version, in which the voxels of the carpet plot were divided into the three tissue classes with voxel ordering based on corresponding delay times as in column (b).

**Figure 3:**
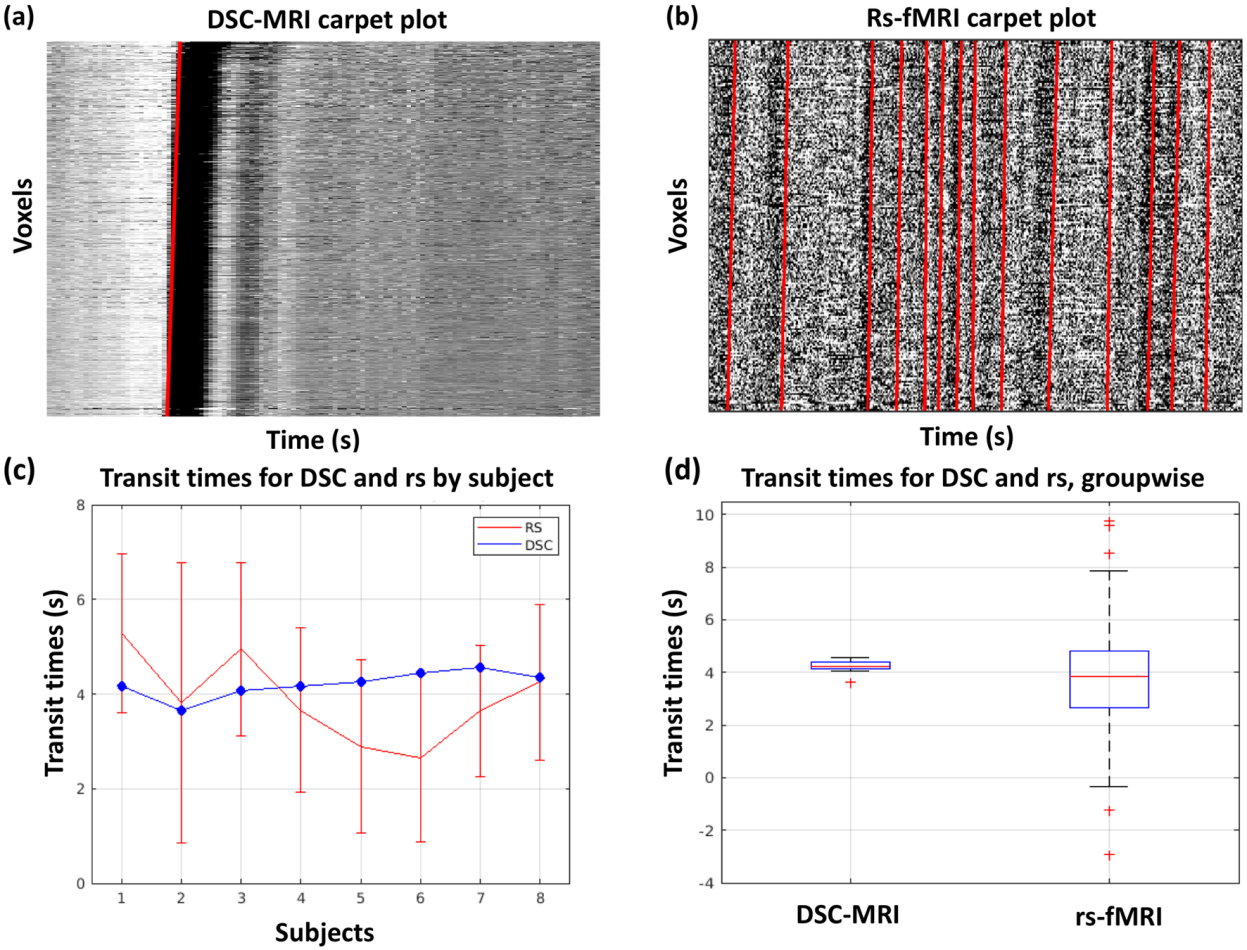
Comparison of edges and transit times computed for DSC and rs-fMRI carpet plots. Carpet plots are displayed for DSC-MRI (**a**) and rs-fMRI (**b**) of the same subject, with identified slopes marked in red lines. Transit times derived from each identified slope for DSC and rs-fMRI carpet plots for all individual subjects are shown in (**c**)*.* The corresponding box plot of the groupwise comparison of DSC and rs-fMRI transit times is shown in (**d**)*.* The 95% confidence interval for the difference between the groupwise medians of DSC-MRI and rs-MRI transit times is [− 0.49, 1.23 s].

**Figure 4:**
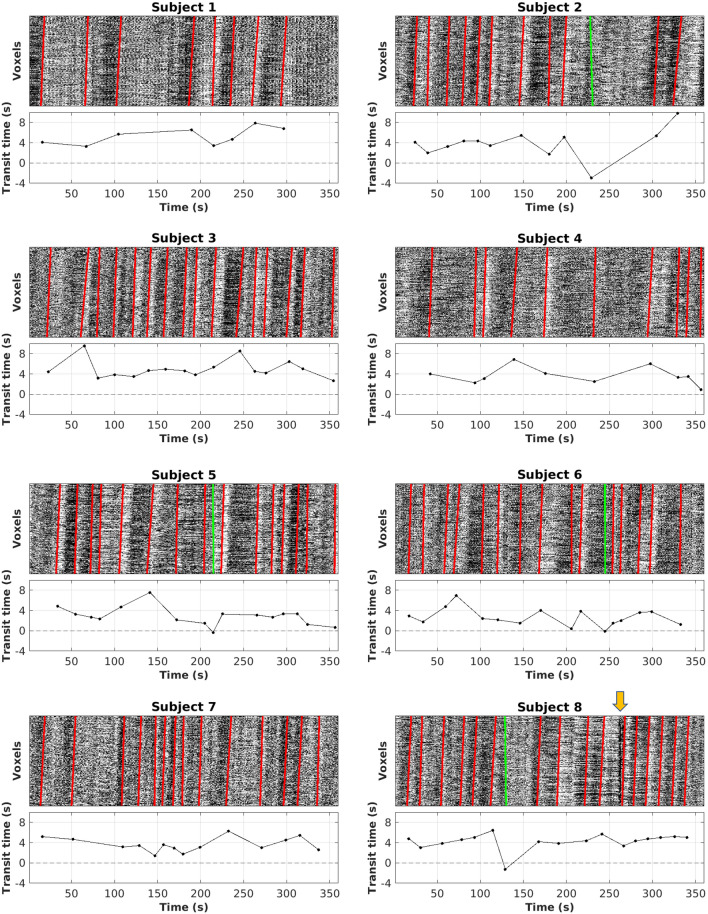
Illustration of the dynamic change in transit time during the rs-fMRI scan for all subjects. For each subject, the rs-fMRI SHAG carpet plot with identified edges is shown on the top, while a plot showing the dynamic change of the transit time of each identified edge is shown on the bottom. Negative transit times indicate an edge sloped in the opposite direction relative to the majority of edges identified; such edges are marked with a green line. Note that for subject 8, we identified a mostly vertical sudden intensity change by the yellow arrow, representing a motion artifact.

**Figure 5:**
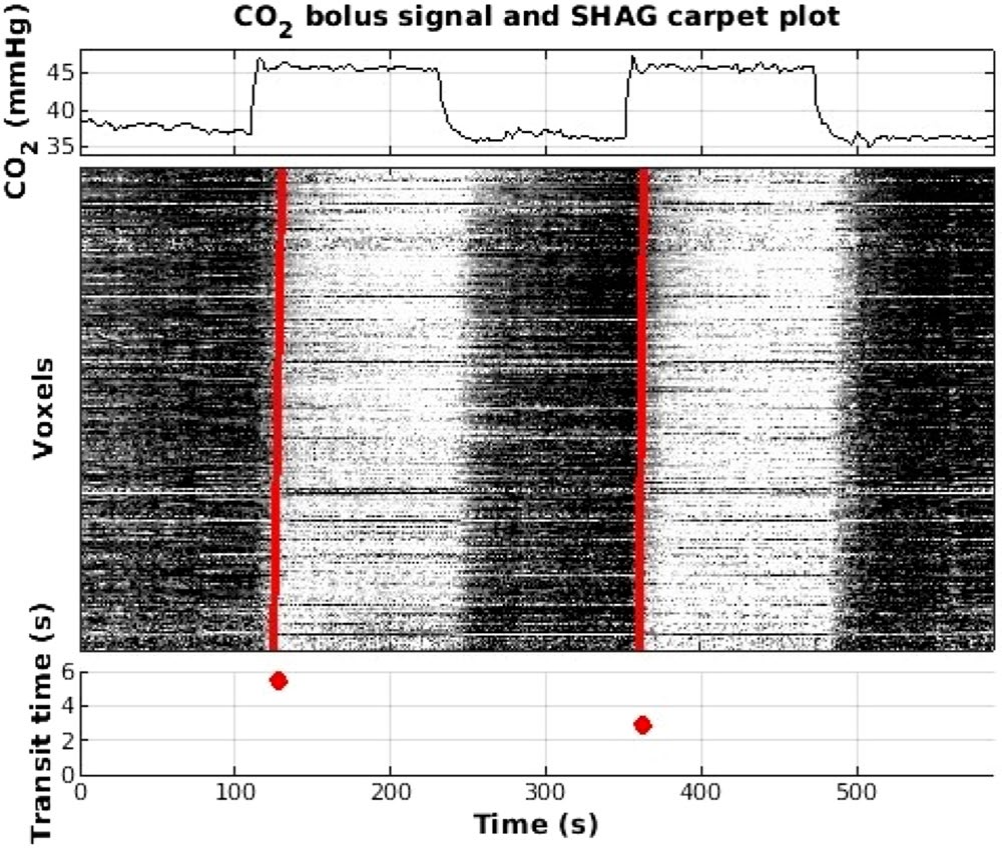
Preliminary example of SHAG carpet plot and transit time computation based on CO_2_ challenge scan. *(Top)* CO_2_ intensity, given in mmHg, administered to subject for inhalation during rs-fMRI scan. *(Middle)* SHAG carpet plot (with noisy voxels cropped) constructed by ordering voxels according to delay times computed via cross correlation of voxel BOLD signal with CO_2_ bolus signal from upper plot. *(Bottom)* Transit times computed for detected slopes corresponding to blood arrival.

A strong motion artifact was present in the rs-fMRI data for the subject displayed in Fig. 2. This artifact (indicated by a yellow arrow) affects all the voxels instantaneously, leading to a vertical line in the carpet plot regardless of the ordering method. Hereafter, all analysis was conducted on the novel method to reorder the carpet plots (column (b) in Fig. 2) with noisy voxels cropped out (as displayed in Figs. 3, 4, and 5).

### Comparing the DSC-MRI and rs-fMRI data transit times

An in-house slope-detection algorithm (see Fig. 1b) was used to detect edges present in each SHAG carpet plot and compute each edge’s transit time. For DSC carpet plots, a single edge was computed which corresponds to the arrival of the gadolinium contrast medium in the brain. This edge is visualized by the sudden drop in BOLD signal intensity (white-to-black edge in plots), marked by a red line in Fig. 3a. For rs-fMRI carpet plots, edges of sudden increased signal intensity (high contrast, black-to-white edges in plots) were detected and marked by red lines in Fig. 3b. Black-to-white edges were chosen for rs-fMRI carpet plots (as opposed to white-to-black edges chosen in DSC carpet plots) because these edges represent increasing signal intensity corresponding to increases in CBF. This is significant because we later analyze SHAG carpet plot edges present in data acquired during a CO_2_ challenge fMRI session. In this CO_2_ challenge analysis, we choose to analyze the edges of increasing signal intensity, since these edges correspond to the arrival of the inhaled CO_2_ bolus (CO_2_ is a vasodilator and causes increased blood flow, resulting in higher BOLD signals^5^). Thus, by choosing to analyze rising-intensity edges in rs-fMRI, we parallel the phenomenon observed in the CO_2_ challenge trial, which will be helpful for future comparisons between the two types of experiments. Further, a groupwise comparison of all rising-intensity edges (109 edges) with all falling-intensity edges (107 edges) from all rs-fMRI subjects was conducted and resulted in no statistically significant difference between the two group means (two sample *t*-test, two-tailed, α = 0.05, *p* = 0.531), suggesting that the choice between the two groups in rs-fMRI would not make a significant difference for the purposes of this study. Rising-intensity rs-fMRI edges were included if they corresponded to time points where the global average BOLD signal exceeded certain thresholds of contrast (change between a local minimum and the following local maximum) and rate of increase in signal intensity (see Methods for threshold values).

The per-subject comparison of edge transit times derived from DSC-MRI and rs-fMRI data is shown in Fig. 3c, while the groupwise comparison is shown in Fig. 3d. The groupwise means and standard deviations of transit time from DSC-MRI (8 edges) and from rs-fMRI (109 edges) are 4.2 ± 0.3 s, and 3.8 ± 2.0 s, respectively. The 95% confidence interval for the difference in median between the two groups (rs-fMRI subtracted from DSC-MRI) is given by [− 0.49, 1.23 s] (computed as described in^26^ for difference of two unpaired samples with approximated normal distribution of *W* statistic).

### The dynamic change of edge slopes and transit times in a single rs-fMRI scan

Transit times were derived from each edge identified in a given rs-fMRI SHAG carpet plot. The dynamic changes in transit time during one rs-fMRI scan are shown in Fig. 4 for all 8 subjects with their corresponding SHAG carpet plots. Notable variations (standard deviation of around 2 s) in transit time during the rs-fMRI scan for a given subject were observed. Note that this variation is not likely due to error in the slope-detection algorithm (see Discussion for evaluation of slope-detection algorithm). However, all rs-fMRI carpet plots show significantly lower SNR compared to DSC carpet plots, as the gadolinium bolus in DSC-MRI produces greater changes in signal intensity than natural fluctuations in the BOLD signal observed in rs-fMRI. Across all subjects, 4 detected edges (out of a total of 109 edges; see green lines in Fig. 4) produced negative transit times, indicating a slope tilted in the opposite direction relative to the majority of edges. Closer examination of the location of these negative transit times showed that they tend to occur at points where multiple faint oscillations occur in close proximity to one another, meaning that a potential explanation for the negative slope is that two oscillations interfered with one another in the slope detection algorithm. Observation of the motion corrections recorded during preprocessing also revealed that some negative transit times corresponded to time points where the subject began a slow head motion, making a motion artifact another potential explanation for the negative transit times, though the exact reason is not yet clear.

### CO2 SHAG carpet plot feasibility

Since sLFOs are spontaneous, the number of identifiable edges in rs-fMRI SHAG carpet plots varies across subjects, leading to uneven assessment of the transit time between different individuals. To solve these uneven assessment limitations, as well as to explore the use of carbon dioxide (CO_2_) as a trackable bolus, we created a SHAG carpet plot using data from a CO_2_ challenge MRI scan (on a new subject), in which respiratory modulation was induced by inhaling elevated CO_2_ during the rs-fMRI scan to “encode” periodic global signal increases into the BOLD fMRI. CO_2_ is a vasodilator, so an increased concentration of inhaled CO_2_ in the bloodstream results in a significant increase in BOLD signal intensity^5^. Voxel ordering for the CO_2_ carpet plot was computed in a manner similar to that of the rs-fMRI data, however here a voxel’s computed arrival time was based on the arrival of the “encoded” CO_2_ bolus signal relative to the measured CO_2_ administration signal (see Methods for details). Fig. 5 displays a preliminary example of the SHAG carpet plot of CO_2_ controlled rs-fMRI data (middle) with the corresponding end-tidal CO_2_ measurement (top). Increases in the administered CO_2_ concentration resulted in increased BOLD intensities in the carpet plot. Edges corresponding to the increase of administered CO_2_ are clearly visible and detectable due to the respiratory modulation, which resulted in the transit times displayed in Fig. 5 (bottom).

### Characterizing sLFOs potentially associated with neuronal activity

**Figure 6:**
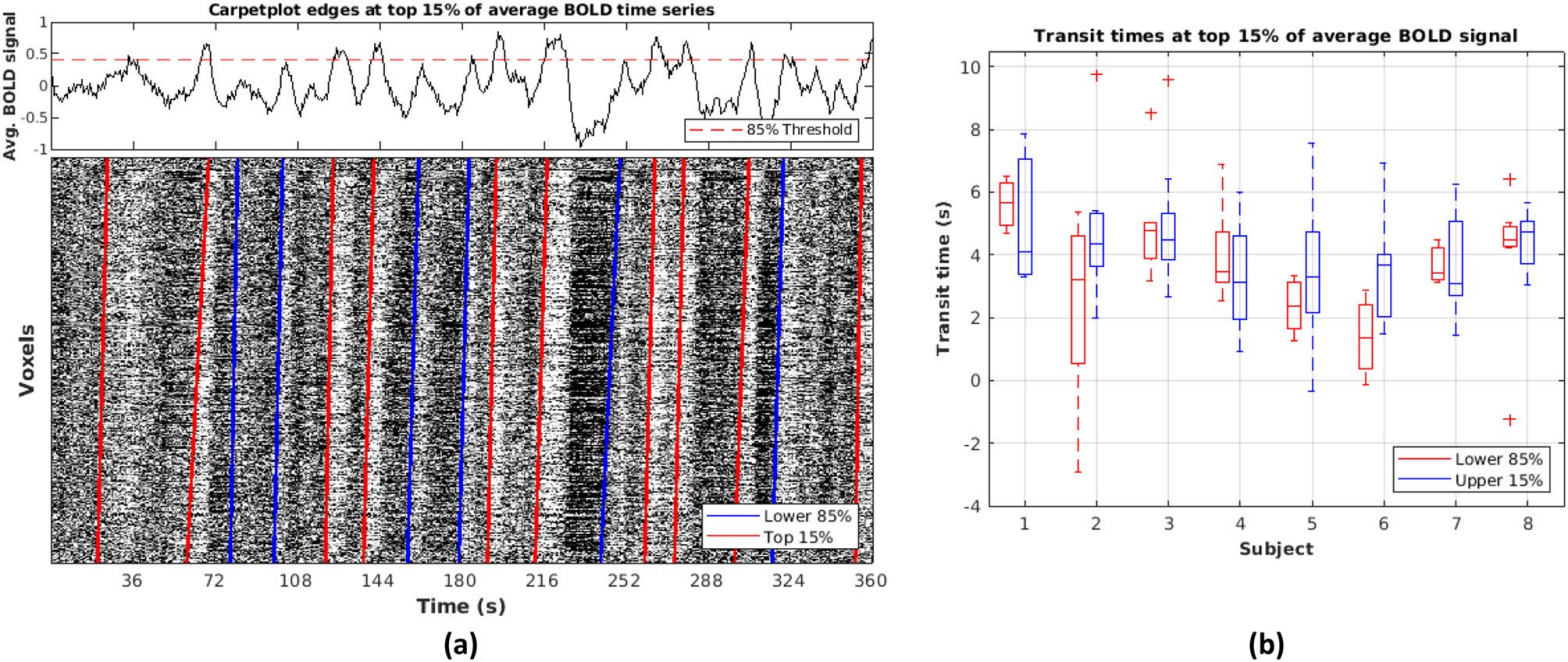
Separation of edges based on predicted neuronal vs. physiological association. (**a**) rs-fMRI SHAG carpet plot edges at top 15% of average BOLD time series. The upper 15% threshold was chosen based on results from Zhang et al.^27^ which implied that fMRI time frames exceeding such a threshold in BOLD intensity may be related to neuronal activity. Edges were detected at rising edges (black-to-white) and designated within “Top 15%” when the edge led into a region within the top 15% of values of the average BOLD signal. (**b**) Comparing transit times for top 15% vs. lower 85% of average BOLD time series. Edges corresponding to the top 15% are those predicted to be rooted in neuronal activity, while the remaining are predicted to be physiological in nature. No significant association was found between computed transit time and assignment to neuronal or physiological categories (mixed effects linear regression model with adjustment for subjects via inclusion as a random factor, *p* = 0.070, computed using Stata statistical software [StataCorp LLC., College Station, TX, version 16, www.stata.com/])^28^.

Figure 6 separates identified edges from rs-fMRI SHAG carpet plots into two groups by ordering the global (averaged across all voxels) BOLD intensity values at each time frame and thresholding the top 15% of global BOLD intensities. This threshold choice of the top 15% of global BOLD intensities was chosen based on a study by Zhang et al.^27^. In this study on a cohort of rats, it was demonstrated that there is a relationship between time points where local field potential (LFP) and BOLD signals are both high in amplitude (upper 15%), implying a neural component of the BOLD signal at these points (more discussion on findings supporting relationships between the BOLD signal and neuronal activity can be found in the Discussion). Thus, edges in the first group (those predicted to correspond with neuronal activity) occur at time points corresponding to the top 15% of global BOLD intensities (total 66 edges), while the remaining identified edges form the second group (total 43 edges). To test the association between transit time and this “neuronal” group classification, a mixed effects linear regression model was constructed (computed and analyzed using Stata statistical software [StataCorp LLC., College Station, TX, version 16, www.stata.com/])^28^ with the described neuronal classification as a fixed factor and subject label as a random factor. The model produced a slope coefficient of 0.665 corresponding to the neuronal classification variable with *R*^*2*^ = 0.025 (*R*^*2*^ computation based only on fixed effects portion of model). No significant association was found between the transit time result and neuronal classification (α = 0.05, *p* = 0.070).

### Evaluating robustness of transit time computations

The slope-detection algorithm was evaluated for robustness by constructing a simple noise model to analyze how consistently the algorithm performed under variation of two parameters: intensity contrast (computed as difference between averaged darkest and averaged lightest carpet plot regions) and noise level. Under the constructed noise model, both DSC-MRI and rs-fMRI SHAG carpet plot images were assumed to consist of two components: an underlying clean image and an added image of Gaussian noise. rs-fMRI SHAG carpet plots produced contrasts in the range of 0.735–1.471 and noise standard deviations in the range of 0.737–0.817. DSC SHAG carpet plots produced contrasts in the range of 2.899–3.226, and noise standard deviations in the range of 0.197–0.330 (see Supplementary Material Sect. 6 for details on these computations). Due to the processing and scaling of the MRI data (see Methods) these measurements are simply in units of signal intensity of the processed carpet plot data.

**Figure 7:**
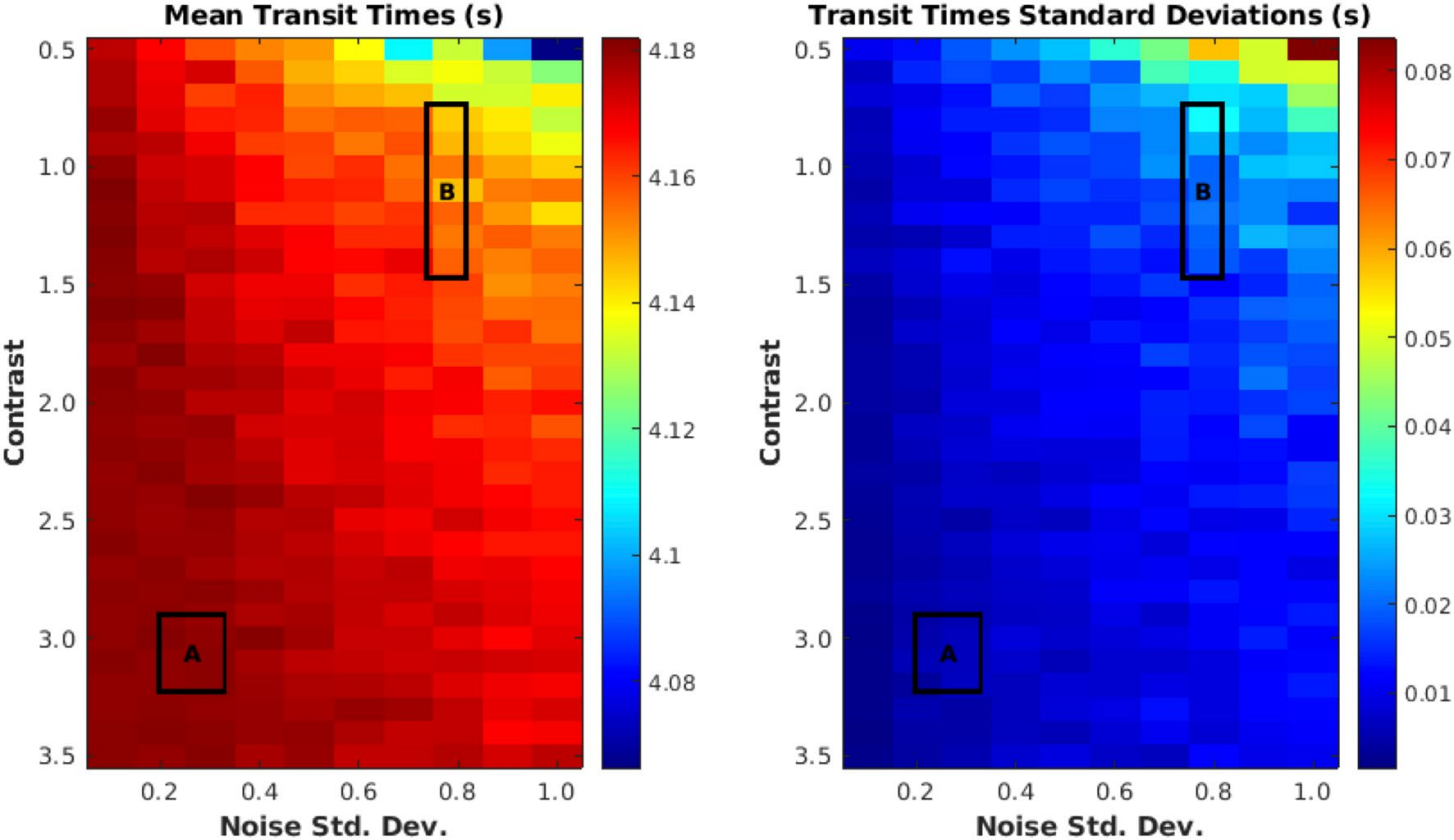
Slope-detection algorithm performance at varying signal contrast and noise levels, given in units of signal intensity resulting after scaling/demeaning of BOLD signals. Contrast reflects the difference between peaks and valleys in the global BOLD signal, while noise level reflects an estimation of the standard deviation of noise assuming a model of additive Gaussian noise. A DSC SHAG carpet plot was scaled to varying contrasts and noise levels (see Supplementary Material Sect. 6) and the transit time of the gadolinium-arrival edge was computed. 30 random white noise trials were conducted at each contrast/noise level pair. Boxes labeled A and B show the regions of contrast and noise level covered by actual DSC-MRI and rs-fMRI SHAG carpet plots, respectively.

This model was developed in order to evaluate the reliability of the slope-detection algorithm, implemented by scaling a chosen DSC carpet plot to varying contrast (ranging from 0.5 to 3.5) and noise levels (ranging from standard deviations of 0.1–1.0) and conducting repeated trials (*n* = 30) of random noise addition at each pairing of contrast and noise level to determine the consistency of transit time computation (see Supplementary Material). The resulting transit time means and standard deviations for this analysis are shown in Fig. 7. We note that decreases in image quality (caused by decreases in contrast or increases in noise level) cause the slope-detecting algorithm to compute lower transit times (higher computed slopes) with higher variability. However, in the lowest quality trial set the transit time was only reduced, on average, by approximately 0.1 s, with a standard deviation of less than 0.08 s.

## Discussion

Power et al.^22^ first proposed a simple but useful way to evaluate fMRI scan quality by using carpet plots. In this study, we extended the application of the carpet plot by proposing and evaluating novel SHAG carpet plots, in which voxels were reordered according to the “arrival time” of the sLFO within each voxel. This method allowed us to study hemodynamics and flow dynamics in the rs-fMRI data by comparing the measurements made using sLFOs with those derived from DSC-MRI; however, it is hard to directly derive the perfusion parameters, such as CBF and CBV, without understanding the true nature of the sLFOs in rs-fMRI. These measurements were taken through the application of an in-house slope-detection algorithm used to detect and quantify edges in SHAG carpet plots. Calculated edge slopes were used to compute an edge’s “transit time”, or the horizontal (i.e. time axis) distance traversed by the edge, which represented the propagation time of some signal pattern throughout the majority of voxels in the brain.

As an injected bolus in DSC-MRI moves through the brain with blood, it should arrive at different voxels at different times, reflected in the “tilted” lines in DSC carpet plots. The slopes of the tilted lines represent the “speed” of blood flow, where larger slopes (steeper tilts) correspond to faster blood flow and shorter transit time. We note that although we fit a linear line to the observed edges, the complexity of blood flow throughout the brain means that a linear fit is likely not a full representation of this flow. However, observation of both DSC-MRI and rs-fMRI SHAG carpet plots indicate that a large portion of the ordered voxels do form a nearly-linear trend, making a linear fit a reasonable starting point for our purposes. In this study, carpet plots were first applied to DSC data to evaluate the sensitivity of our edge/slope-detection algorithm and whether the resulting transit times match previous studies. DSC carpet plot edges produced transit times at 4.2 ± 0.3 s (see Fig. 3c), which aligns reasonably well with literature values for cerebral circulation time (CCT) using ultrasonography methods. Hoffmann et al.^29^ reported CCT of 5.6 ± 1.7 s with Levovist as the contrast agent in 25 healthy volunteers, while Liu et al.^30^ reported CCT of 6.3 ± 1.3 s for the start time with the contrast agent SonoVue in 67 healthy subjects. These CCT values are longer than the transit times derived here from DSC-MRI SHAG carpet plots. This is likely due to the fact that in these studies CCT was measured as the interval of flow between the internal carotid artery and the internal jugular vein, while our values only represent the transit time through the brain.

The rs-fMRI SHAG carpet plot edges produced transit times in a similar range, though with higher variance (4.2 ± 0.3 s for DSC, and 3.8 ± 2.0 s for rs-fMRI; 95% confidence interval of [− 0.49, 1.23 s] for difference between group medians), compared with those from DSC carpet plots in a groupwise comparison (see Figs. 3c,d). This indicates that the “flow” of the sLFOs bears some similarity to the bolus flow (DSC) in terms of transit time^10^. Given the fact that spatial smoothing will mix signals in voxels with their neighborhood voxels, potentially mixing sLFOs with other signals (e.g., Mayer waves), we compared the rs-fMRI SHAG carpet plots and their corresponding transit times with and without spatial smoothing applied. The spatial smoothing was ultimately chosen for the following reasons: (1) improved signal-to-noise ratio; (2) fewer voxels are discarded; (3) no change to the dynamic pattern of the transit times and the transit time values on the full, GM, WM, and CSF only carpet plots (see Supplementary Fig. S4 and Fig. S5).

**Figure 8:**
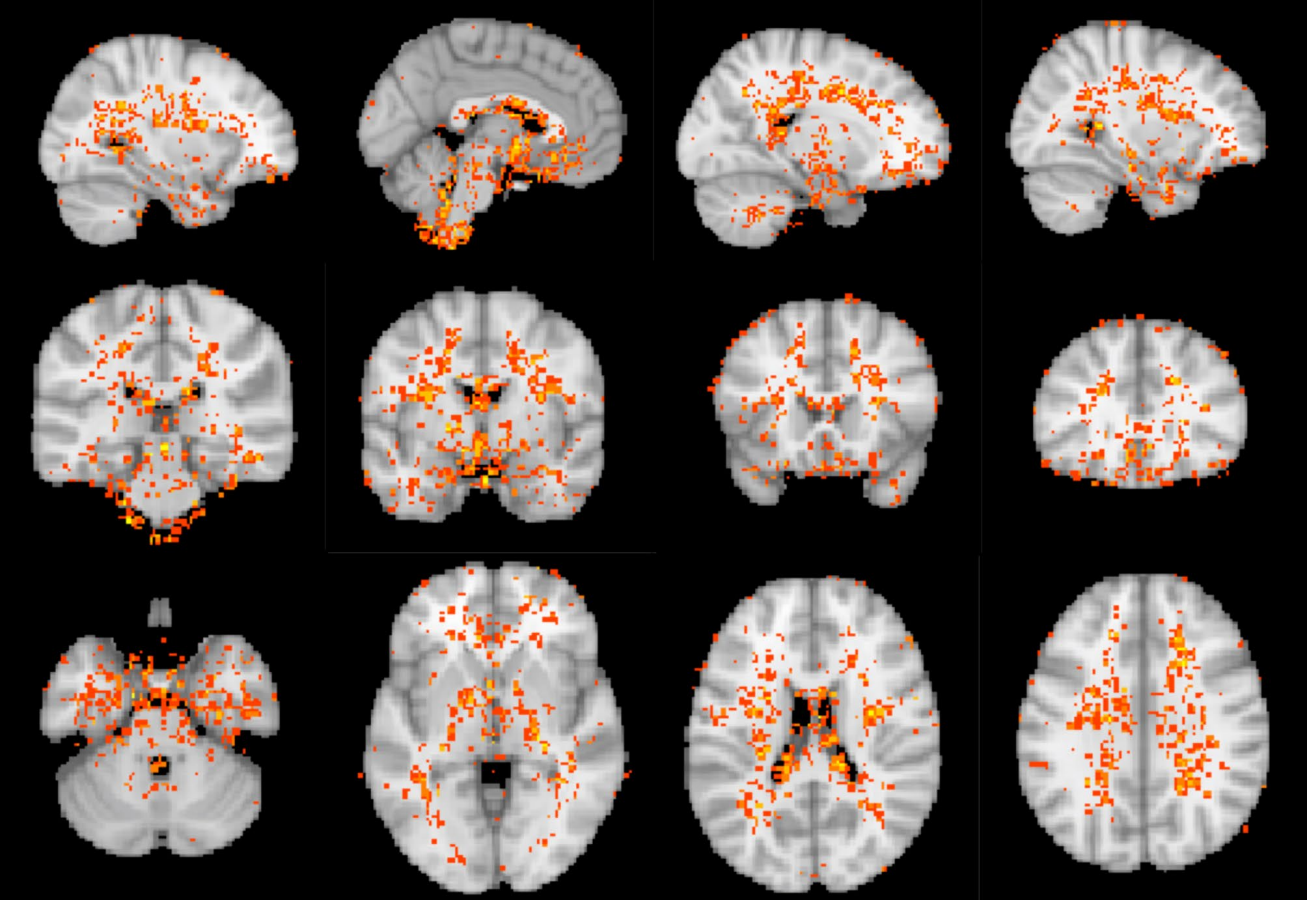
The averaged spatial map of removed voxels with the standard brain as overlay. Voxels that were removed in at least half of the subjects were considered as voxels that were commonly removed among this set of subjects and shown in this map.

On average, about 29% of voxels in the rs-fMRI scans were discarded before slope detection due to the unclear display of the pattern of sLFOs (see Supplementary Fig. S2). No one tissue type dominated these voxels; 17% of GM voxels, 16% of WM voxels, and 8% of CSF voxels were discarded (see the histograms in Supplementary Fig. S3). Only about 7% of the removed voxels fell on the interfaces between GM, WM, and CSF. The majority of the removed voxels reside in or around multiple ventricles and in the deep gray matter (Fig. 8) where the sLFOs are low due to the lack of blood vessels. Thus, the transit times of sLFOs in rs-fMRI SHAG carpet plots may be underestimated, which implies that the actual transit times of sLFOs are likely longer than the transit time of the bolus in DSC scans.

Even though carpet plots cannot show the dynamic pattern of the contrast bolus or sLFOs moving through the brain, these dynamic patterns have been found to be largely consistent with a positive relation between the delay maps from rs-fMRI and TTP maps from DSC-MRI (see Fig. S7)^10^. As a result, sLFOs have been used as a natural bolus to track blood flow in the brain^31^. It was recently shown that the delay time between the sLFOs extracted from BOLD signals at the internal carotid artery and the superior sagittal sinus (SSS) were about 5 s in 100 healthy adults^31^. However, discrepancy was observed between the delay map from the rs-fMRI and the TTP map from the DSC-MRI (see Supplementary Fig. S8). Previous work gave possible explanations, which are (1) heterogenous effects of the imaging contrast [i.e., Hb] in rs-fMRI while homogenous effects of the imaging contrast (i.e., Gd) in DSC-MRI in different vessel types (i.e., arteries, veins, and capillaries); (2) partial volume effect on rs-fMRI only; (3) different sensitivity to the blood flow between rs-fMRI and DSC-MRI (please refer to our previous work for details)^10^. Additionally, some studies have found that rs-fMRI signals are encoded by the arterial CO_2_ concentration^32,33^ coupled with regional specific hemodynamic response to CO2 fluctuations^34^. These physiological reasons above increase the complexity of the delay time from the rs-fMRI using sLFOs, leading to discrepancies between the rs-fMRI using the sLFOs and TTP from DSC-MRI.

Moreover, we would like to expand on the explanation of the differences between the flow of sLFOs and gadolinium bolus based on recent findings of neuronal components in LFOs (i.e., global signal of fMRI). It has been suggested with growing evidence that the global mean fMRI signal contains contributions from not only “nuisance” components (e.g., physiological drift, motion, cardiac, respiratory, and vascular components), but also neuronal components^35^. Chang et al. found a significant negative correlation between the local field potential measures of brain arousal on macaque monkeys with the global mean fMRI signal^36^. Later, it was found by the same group that this widespread signal is closely related with momentary drops of brain arousal in subcortical regions on data from the Human Connectome Project^20^. Moreover, a study in mice has shown that neuronal signals are embedded in the global waves that propagates across the entire neocortex^37^. In this study, the neuronal signal was directly measured through calcium signals using wide-field optical imaging. These findings indicate the existence of propagating global neuronal signals. It has been demonstrated by Liu et al.^38^ that when using only the top 1 to 15% of averaged resting state BOLD time frames from the posterior cingulate cortex and medial prefrontal cortex, connectivity patterns are derived which resemble functional connectivity networks, indicating that the functional networks may be driven by simultaneous brain activities. Recently, Zhang et al.^27^ also provided more direct evidence by concurrently examining rs-fMRI and taking local field potential (LFP) measurements in rats. They found that 70–100% of positive BOLD events are preceded by an increase in LFP power. In addition, they reported that averaged fMRI time frames with the highest 15% of LFP power and BOLD intensity accurately resemble the spatial pattern of the LFP and BOLD signal cross-correlation map, which demonstrates a relationship between when these two signals are high in amplitude, and implies a neural underpinning for the BOLD signal. In our study, we applied these findings to better understand the relationship between neuronal activity and sLFOs using SHAG carpet plots. We hypothesized that the propagations of neuronal signals might be faster than those of vascular origins. We explored the propagation of LFOs with high magnitude (i.e., more relevant to neuronal signal) and those without (relevant to physiological changes) by thresholding the top 15% (per the upper threshold described by Zhang et al.^27^) of time frames in the global fMRI signal. However, our results indicate no conclusive difference in the transit time between these two categories.

We also explored the application of our method to assess blood flow speed moment by moment during rs-fMRI scans. Compared to other methods, a rs-fMRI SHAG carpet plot offers a simple and more direct graphical representation of the dynamic propagation of sLFOs in the brain during the scan. Moreover, rs-fMRI SHAG carpet plots present the potential to assess this propagation (i.e., transit time) at different time points (Fig. 4) during the scan, which may indicate variations in the speed of blood flow. In fact, results from the evaluation of the robustness of the transit time computation (Fig. 7) indicated that data noise at the scale present in these SHAG carpet plots produced relatively small variations in the transit time computation, supporting the notion that the variations in rs-fMRI transit times within a single scan are due to true underlying variations and not simply results of sensitivity to noise. This is a huge advantage over methods based purely on cross-correlation, the result of which is an averaged transit time across the duration of an fMRI scan. However, sLFOs are spontaneous and their amplitudes vary dramatically among subjects, meaning that the number of detectable edges and their temporal locations vary across subjects. This leads to difficulties in drawing comparisons between individuals. Also, rs-fMRI carpet plots suffer from low SNR, which may affect the accuracy of slope detection. One solution for these limitations is to “encode” large periodic signals in the BOLD fMRI signal. This may be accomplished by introducing respiratory modulation through elevated CO_2_ levels. As shown in Fig. 5, periodic edges with improved contrast were introduced into the carpet plot. This modulation can be consistently introduced across subjects, improving comparability. To assess the arrival time as done in DSC, the CO_2_ SHAG carpet plot’s rising (black-to-white, or that correspond to the rise of the end-tidal CO_2_ curve) edges were analyzed. The transit times are 5.42 and 2.94 s for the two rising edges in this preliminary example. We note that the transit time is similar to those of DSC. Moreover, the variation in transit time may be due to the fact that CO_2_ is a potent vasodilator, which causes complicated hemodynamic changes among voxels. These hemodynamic effects will require more exploration as this method is studied further.

The slope detection algorithm was evaluated to see how changes in image contrast (which reflects SNR of oscillations in data) and image noise levels affects the computation of transit times. The results of this analysis are shown in Fig. 7. This analysis indicated that decreasing the strength of the underlying oscillation signal, either by decreasing contrast (reflecting SNR) or increasing noise levels, tends to cause the computed transit time to decrease, though this decrease (average decrease 0.1 ± 0.08 s) was relatively low even in the lowest quality trial set, which simulates images of lower quality than the actual DSC-MRI and rs-fMRI carpet plots under the described noise model. The variation in transit time examined throughout this study is often of much greater magnitude than these reported decreases, implying that such variations stem from underlying structural differences in the carpet plots as opposed to the algorithm’s sensitivity to noise. This observation is particularly useful in interpreting variations in edge transit time within a single rs-fMRI scan (Fig. 4), implying true underlying differences between the slopes of edges across the scan time.

We also note that the method for computing transit times will contain some degree of error because of the temporal resolution limitations imposed by the TR of the imaging sequence. This potential error can be thought of as a function of three parameters: (1) the true underlying transit time, (2) the TR of the imaging sequence, and (3) the time point where sampling begins. The magnitude of the potential error is inversely proportional to the true underlying transit time. In the worst case, where the true transit time is smaller than the TR, then the computed transit time could have an error of up to the magnitude of the TR. However, given that we generally expect transit times in the range of 4–6 s and the largest TR used was 1.5 s for DSC-MRI, we can expect errors much lower than the value of the TR. A simulation was run applying the slope detection algorithm to artificial carpet plot edges, varying the true underlying transit time and time point at which sampling began with a fixed TR (this is discussed in more detail in the Supplementary Materials Sect. 5). For instance, it was found that for a true underlying transit time of 4.5 s, with a TR of 1.5 s, we can expect errors ranging between − 0.6 and 0.3 s. Given that, for the purposes of this analysis, the true transit time and sampling start time will vary randomly between real carpet plot edges (thus randomly varying the error), we expect that groupwise comparisons of edges will mostly remove the effects of this source of transit time error, but could result in slightly increased variance and slightly underestimated transit times. We do not expect these errors to be strong enough to significantly affect the results of groupwise comparisons of DSC-MRI and rs-fMRI transit times presented in this study. Further, the within-subject variation of rs-fMRI transit times between edges is much greater than the potential error described here, meaning that these variations likely represent true differences in propagation speeds as opposed to just reflecting algorithm errors.

## Limitations

Though our analysis showed consistency between transit times computed for rs-fMRI and DSC carpet plots, the spatial flow dynamics cannot be reflected by a 2D carpet plot only. The discrepancy between the flow dynamics needs further evaluation to better understand the relationship of sLFO slopes with blood flow. In addition, the chosen method for ranking rs-fMRI voxels relies heavily on the global BOLD signal, which has the consequence of potentially ignoring regional signals more heavily related to resting-state networks. We note that on average, about 29% of voxels (which reside in WM, GM, and CSF) in the rs-fMRI scan were discarded for the slope detection since the sLFOs in those voxels are low due to the lack of blood vessels. This could lead to underestimation of the transit time if the voxels at the starting and ending regions of the travel path were discarded as noise voxels. We also note that the noise model used to evaluate the slope-detection algorithm represents an oversimplification of the noise observed in rs-fMRI data. The noise which corrupts the BOLD signal is not simply Gaussian in nature, and it may not be ultimately correct to assume that perfectly linear underlying edges exist beneath the noise in carpet plot images. This limits our ability to yet claim causal physiological differences between edges of different transit time. Finally, the temporal resolution limitations of the imaging sequence TR affect the accuracy of transit time computations, as explained in the Discussion section.

## Materials and methods

### Data acquisition

All experimental protocols used in this study were approved by the Institutional Review Board (IRB) of the institution at which the datasets were conducted (McLean Hospital for DSC-MRI and rs-fMRI, Purdue University for CO_2_ challenge MRI) and were compliant with the ethical principles of the Belmont Report. All subjects provided written informed consent. DSC-MRI and rs-fMRI scans of eight healthy subjects (1F, 7 M, mean ± s.d., 33 ± 12 years) were acquired on a Siemens TIM Trio 3 T scanner (Siemens Medical Solutions, Malvern, PA) with 32-channel phased array head matrix coil. The rs-fMRI scan (TR/TE = 720/32 ms, voxel size = 2.5 × 2.5 × 2.5 mm^3^, duration = 360 s) was followed by the DSC-MRI scan (TR/TE = 1510/21 ms, voxel size = 1.8 × 1.8 × 3.5 mm^3^, duration = 180 s) for each subject. In DSC scans a gadolinium contrast medium was given by intravenous injection. Detailed acquisition information can be found in the previous publication by Tong et al.^10^.

CO_2_ challenge data for a separate subject were obtained on a 3 T GE Discovery MR750 MR scanner (TR/TE = 1000/30 ms, voxel size = 3 × 3 × 3 mm^3^, duration = 600 s). The CO_2_ challenge was given via the programmable computer-based gas delivery system RespirAct (Thornhill Research Inc. Toronto, Canada)^39^. The breathing protocol was set as follows: two blocks of CO_2_ of 10 mmHg higher than baseline with 2-min baseline before, in between and after the CO_2_ stimuli. The end-tidal CO_2_ timeseries were measured by this system.

### Data pre-processing

All data were preprocessed using the FMRIB Software Library (FSL, Oxford University, UK, v5.0 for DSC and rs-fMRI data, v6.0 for the CO_2_-MRI data, https://fsl.fmrib.ox.ac.uk/fsl/fslwiki)^24^, including motion correction, slice-time correction, brain extraction, and spatial smoothing (3 mm for DSC and rs-fMRI scans, 5 mm for CO_2_-MRI scan). Given the different scan parameters between the DSC-MRI scans and rs-fMRI scans (e.g., voxel sizes, FOV), the DSC-MRI images were registered into the space of rs-fMRI images for the purpose of comparison between these two.

### SHAG carpet plot construction

Carpet plot construction and all further analysis was completed using MATLAB (version R2017b or later, www.mathworks.com/). SHAG carpet plots were generated by reordering timeseries of voxels based on their relative delays. Three different methods were implemented to calculate the relative delays for DSC-MRI, rs-fMRI, and CO_2_-MRI.

The voxel-specific delay for DSC-MRI data was represented by time to peak (TTP), which is defined as the duration between the beginning of the scan and the maximum dip of the DSC signal loss. TTP was calculated by the program Perfx using a gamma function fitting with temporal interpolation (developed by Chris Rorden, www.mccauslandcenter.sc.edu/CRNL/tools/pwi). The new temporal resolution for TTP is 0.0001 s.

The voxel-specific delay for rs-fMRI was represented by the relative delay between the sLFO of BOLD signal from the global mean signal (as a reference) to any given voxel. For computation of the delay time, the BOLD signal was interpolated with a new temporal resolution as 0.072 s. The sLFO in each voxel’s BOLD signal was isolated using a band-pass filter (0.01–0.1 Hz, zero-lag 4^th^-order Butterworth filter). The delay time for each voxel was calculated by performing a cross-correlation between each voxel’s sLFO and the averaged sLFO across all voxels in the brain. The time corresponding to the maximum cross correlation coefficient (MCCC) formed the relative delay for that voxel. We note that even after motion correction, there is an obvious motion artifact observed in one subject’s rs-fMRI SHAG carpet plot (see Fig. 2). The effect of a single motion artifact on the accuracy of the delay time calculated is limited (see Supplementary Fig. S6).

The voxel-specific delay of CO_2_-MRI data was determined by measuring the relative delay between the very low frequency oscillation (1/240 Hz) of BOLD signal, due to the CO_2_ challenge, and the independent end-tidal CO_2_ measurement (as a reference). Specifically, we obtained the CO_2_-modulated BOLD signal using a bandpass filter (0.001–0.02 Hz) and performed the same cross-correlation between the filtered BOLD signal and the end-tidal CO_2_ timeseries. Interpolation was used to increase the temporal resolution of the CO_2_ modulated BOLD signal from the original TR of 1 s to a new resolution of 0.1 s. The same was done for the end-tidal CO_2_ measurement signal, meaning that the computed delays had a temporal resolution of 0.1 s.

All carpet plots were constructed by vertically concatenating the scaled, demeaned, unfiltered BOLD MRI signal/DSC signal for all voxels based on their corresponding delay times in a descending order**.** We note that some voxels do not show clear and consistent patterns similar to the majority of the voxels in a carpet plot. These voxels were likely stacked at the top and the bottom of the carpet plot due to the extremely long delay times (0.8 ± 0.7 (s.d.) % of all voxels for DSC, 28.7 ± 5.2 (s.d.) % for rs-fMRI, see Supplementary Fig. S1 and Fig. S2) with weak MCCCs. We cropped (removed some continuous portion from the top and/or bottom of the matrix) the SHAG carpet plots such that only those voxels which shows clear and consistent patterns remained, forming the cropped SHAG carpet plots used throughout the rest of the paper. GM, WM, and CSF masks were generated by MATLAB-based software (v1.0, http://doi.org/10.5281/zenodo.3871362)^25^ in order to create the segmented rs-fMRI SHAG carpet plots.

### Slope-detection algorithm

The slope-detection algorithm detects a selected *n* number of edges in a given carpet plot image (Fig. 1b, i) and computes each edge’s corresponding slope (steepness of the tilt in the edge) and estimated time duration traversed by the edge (time difference between the bottom and top of an identified edge), denoted as “transit time” (see Fig. 1b, v). The slopes of the edges can provide information about the “speed” of blood flow, where larger slopes (steeper tilts) correspond to faster blood flow and shorter transit time.

The algorithm first smooths the image via a 2D blurring filter to reduce noise (Fig. 1b, ii). The time locations of the edge centers are then estimated by determining the *n* locations of highest derivative value peaks for the timeseries averaged over all voxels. The smoothed carpet plot image is then filtered with a 2D horizontal derivative filter (Fig. 1b, iii). For each estimated edge location, a selected range of columns immediately surrounding the time location (see Supplementary Material Sect. 5 for selection of range) is observed. For each row (voxel) in this observed range, the column (time) location of the highest value of the row from the derivative-filtered carpet plot image is recorded. This forms a set of coordinates (Fig. 1b, iii) which are used to fit a linear regression line that estimates the edge seen in the original carpet plot image (Fig. 1b, iv). Please refer to the Supplementary Material for further details regarding the slope detection algorithm transit time computation (Sects. 4 and 5, respectively).

### Data analysis

To understand the dynamics of the sLFOs in the brain during rs-fMRI, edges of the sLFOs in the rs-fMRI SHAG carpet plot were identified as follows: the slope-detection algorithm first detected up to 36 rising-intensity edges in each rs-fMRI carpet plot; the value 36 was selected because it represented the upper bound of possible edges found in a single rs-fMRI SHAG carpet plot based on the duration of a rs-fMRI scan (360 s) and the frequency of oscillations (highest frequency 0.1 Hz), though many fewer edges were actually included by the developed algorithm due to the selected constraints. Of the maximum possible 36, only edges corresponding to global BOLD signal contrast (computed for each detected edge as the difference between the preceding minimum peak and the following maximum peak relative to the estimated edge location) exceeding 0.2 in signal intensity were kept for consideration, resulting in 8–17 edges (mean ± s.d., 13.6 ± 3.3) per subject. For DSC carpet plots, only one falling-intensity edge, corresponding to the arrival of the gadolinium bolus, was evaluated (Fig. 2, lower row). Means and standard deviations of transit time were calculated for all rs-fMRI SHAG carpet plots, and the resulting transit times were compared with DSC-MRI Shag carpet plot transit times both within and across all subjects (Fig. 3). A Wilcoxon rank sum test framework (as described in^26^ with the *W* statistic approximated as normally distributed) was used to construct a 95% confidence interval for the difference in median transit time of DSC-MRI and rs-fMRI SHAG carpet plots.

To assess the transit time during CO_2_ challenge scans, edges were detected for the major rising edges of the administered CO_2_ challenge SHAG carpet plot (two rising edges).

Transit times for each edge (8–17 edges in rs-fMRI and 1 in DSC, see Figs. 3 and 4) were computed. To assess the transit time of edges with predicted neuronal and physiological origins, the edges detected in rs-fMRI SHAG carpet plots (as described previously) were separated into two groups, the first group containing edges directly preceding a peak in the global average BOLD signal which fell within the top 15% of all average BOLD signal time frames (5–11 edges per subject), and the second group containing the remaining edges (3–8 edges per subject). A mixed effects linear regression model (neuronal group assignment as a fixed factor, subject as a random factor) was computed using Stata statistical software (StataCorp LLC., College Station, TX, version 16, www.stata.com/)^28^ to evaluate the association between transit time and group assignment.

## Supplementary Information

Supplementary Information

## Data Availability

Code and sample data are available on Github at the following link: https://github.com/TonglabPurdue/Carpetplot.
