# Supplementary material for: Using carpet plots to analyze transit times of low frequency oscillations in resting state fMRI
Source: Sci Rep. 2021 Mar 26;11:7011. doi: 10.1038/s41598-021-86402-z (PMC7998022; doi:10.1038/s41598-021-86402-z)
Supplement: Supplementary file 1 — Supplementary Information [file 41598_2021_86402_MOESM1_ESM.pdf]

Supplementary Material for

Using carpet plots to analyze transit times of low frequency oscillations in resting state fMRI

Bradley Fitzgerald[2], Jinxia (Fiona) Yao[1], Thomas M. Talavage[1,2], Lia M. Hocke[3], Blaise deB Frederick[3] and *Yunjie Tong[1]

[1]Weldon School of Biomedical Engineering, Purdue University, West Lafayette, IN, 47907, USA
[2]School of Electrical and Computer Engineering, Purdue University, West Lafayette, IN, 47907, USA
[3]Mclean Hospital, Harvard Medical School, Belmont, MA, 02478, USA

***Corresponding Author:**
Yunjie Tong
Address: 206 S. Martin Jischke Drive, West Lafayette, IN 47907-2032, USA
Tel: 765-494-0198
Email: tong61@purdue.edu

1. Full SHAG carpet plots for DSC-MRI and rs-fMRI

SHAG carpet plots, when first reordered, tend to result in voxels grouped in the upper or lower portions of the plots which do not contain signal patterns consistent with that seen in the majority of voxels (See Fig. S1 for full SHAG carpet plots). These grouped voxels were removed from each carpet plot in order to perform a linear fit to edges present in the plots (See Fig. S2 for percentage of voxels removed). The distribution of discarded voxels in GM, WM, and CSF are shown in Figure S3.

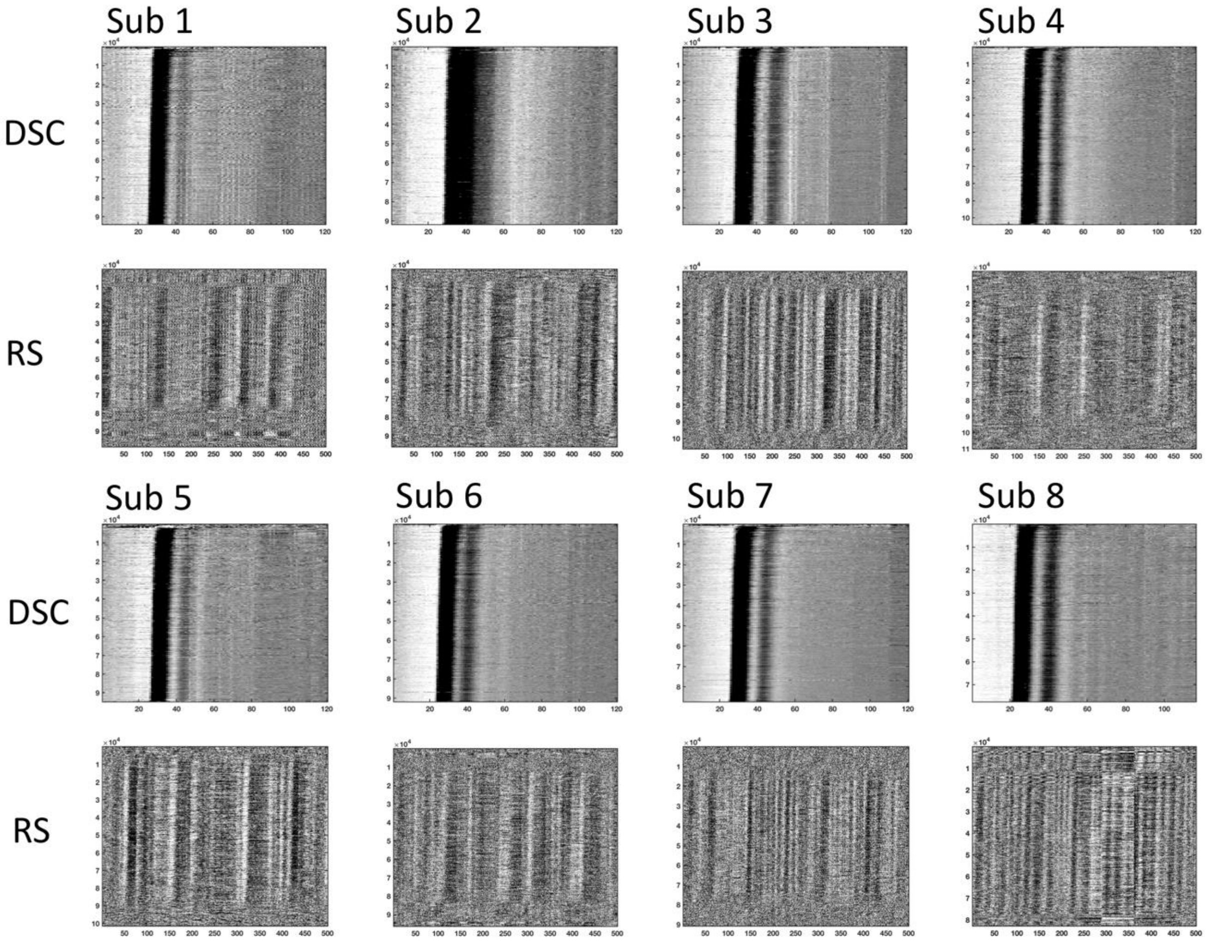

Figure S1: The full SHAG carpet plots for DSC and rs-fMRI for eight subjects. The DSC-MRI SHAG carpet plots are shown in the first and third rows while the corresponding rs-fMRI SHAG carpet plot are shown in the second and fourth rows.

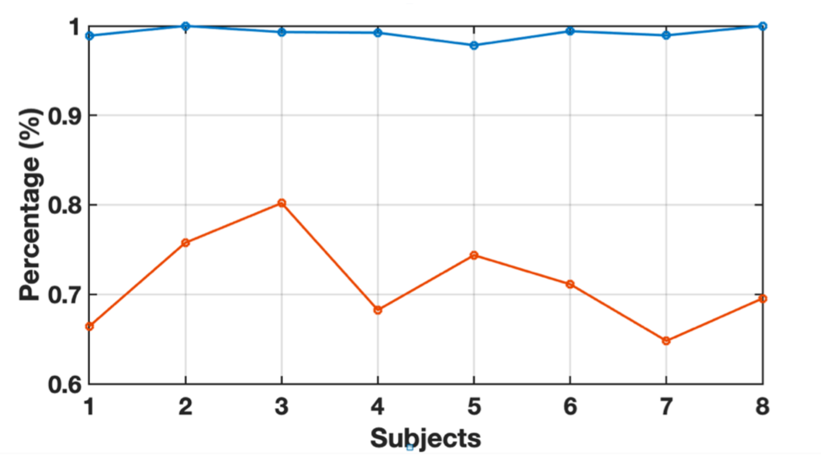

Figure S2: The percentage of cropped portion of carpet plots for the analysis throughout the paper. 99.2 ± 0.7 % of all voxels for DSC (blue) and 71.3 ± 5.2 % for rs-fMRI (red) are used.

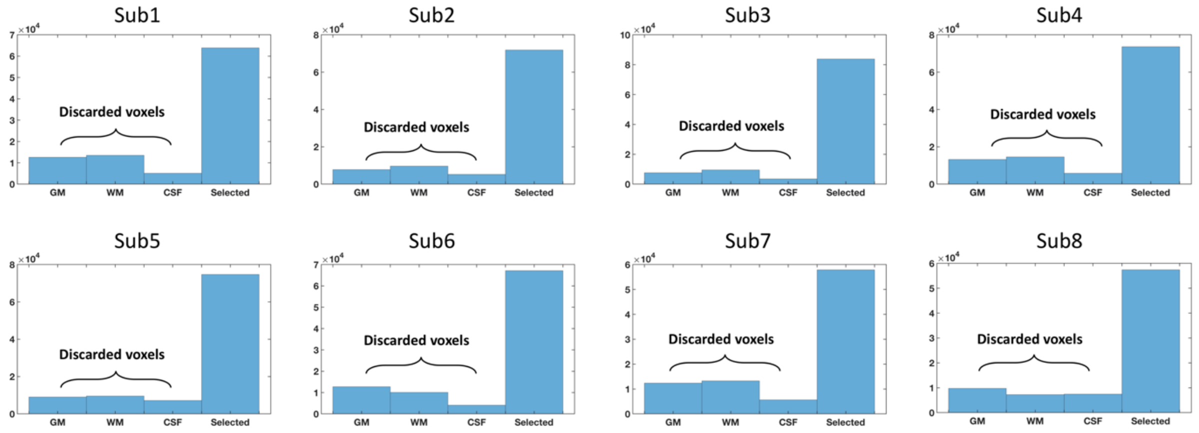

Figure S3: A histogram for each subject presenting the distribution of discarded voxels in GM, WM, CSF, and the selected voxels in total. The means and the standard deviations of the percentage of discarded voxels in GM, WM, and CSF were 17%±6%; 16%±5%; 8%±3%, respectively.

2. The effects of spatial smoothing and motion artifact on the transit time and delay time calculation

Given the fact that spatial smoothing will mix signals in voxels with their neighborhood voxels, potentially mixing sLFOs with other signals (e.g., Mayer waves), we compared the rs-fMRI SHAG carpet plots and their corresponding transit times with and without spatial smoothing being applied. Signal-to-noise ratio was improved and fewer voxels were discarded when the spatial smoothing was applied (See Fig. S4). There were no significant differences on the transit time in cropped carpet plots in full, GM, WM, and CSF, respectively (ANOVA test). Dynamic change patterns within the subject also remain very similar (See Fig. S5). For these reasons, it was determined that spatial smoothing is still necessary in the preprocessing step.

Even after motion correction, there is an obvious artifact observed in one subject's rs-fMRI SHAG carpet plot (See Fig. 2). A simulation was done to evaluate the effect of a single motion artifact on the delay time calculation (See Fig. S6). In the simulation, the original BOLD was shifted by 3 seconds to form a new BOLD signal (shifted BOLD). The head motion was added to both signals at the same time point (See Fig. S6(A)). The size of the signal spike due to the head motion was simulated as 0 to 5 times (with 0.1 increment) the standard deviation of the given BOLD signal. After the preprocessing steps, signal interpolation and filtering used in the paper, cross-correlation was performed between the sLFOs from both original BOLD and the shifted BOLD. The resulting delay times and the corresponding maximum cross-correlation coefficients (MCCCs) were shown in Figure S6(B). The MCCC values are very high in all cases (0.970-0.975). The corresponding delay times did deviate from the true value (3s), which are 2.9 s (0-4.9 times the SD) and 2.8 s (5 times the SD). Even with the large motion artifact (5 times the SD), the deviation is within 0.2 s. Therefore, we demonstrated that head motion occurring instantaneously across the whole brain had limited effects on the delay time calculations.

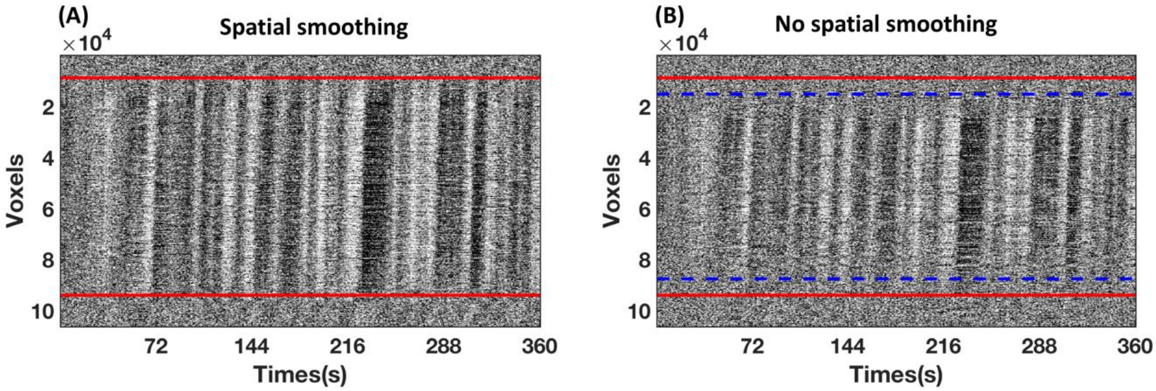

Figure S4: Carpet plots with/without spatial smoothing being applied. Voxels between the red lines are the cropped portions with spatial smoothing (A), while voxels between the blue lines are the cropped portion without spatial smoothing (B).

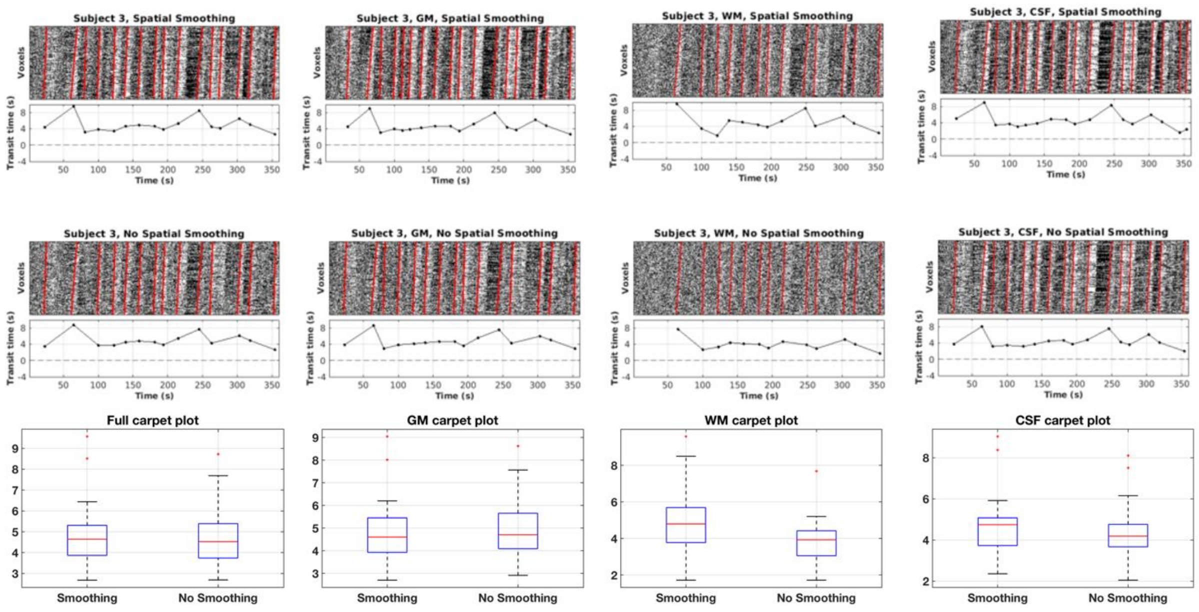

Figure S5: The comparisons of transit times computed from cropped carpet plots in full, GM, WM, and CSF only with and without spatial smoothing.

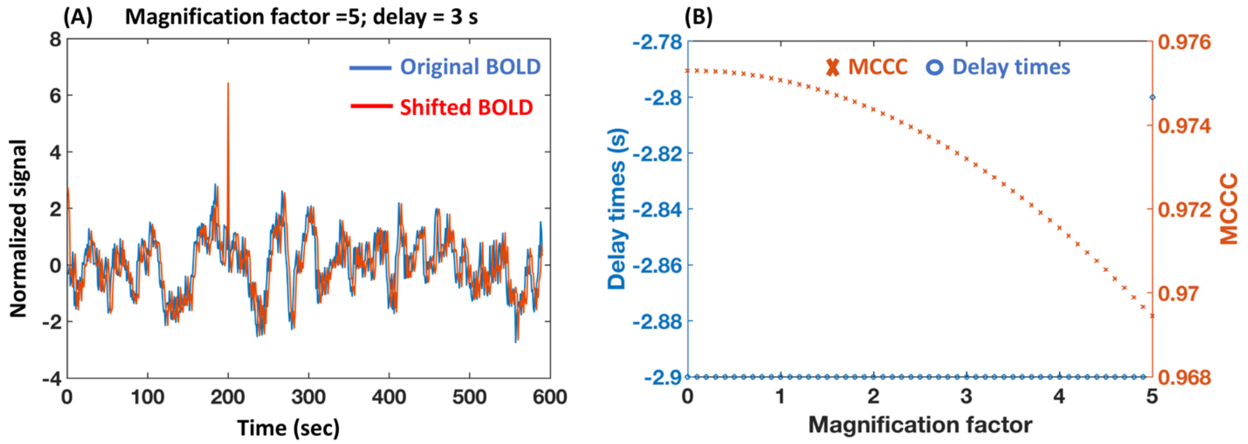

Figure S6: A simulation evaluating the effect of a single motion artifact on the delay time calculation. (A) shows simulated original BOLD (in blue color) and shifted BOLD (in red color; shifted by 3 sec) with the motion artifact occuring at the same time (the magnification factor of the motion spike is 5 times the standard deviation of the original BOLD without motion artifact). (B) displays the cross-correlation results between the original BOLD and shifted BOLD with the magnification factors from 0 to 5 times the standard deviation of the original BOLD, the delay time in seconds (blue circle) and the corresponding maximum cross-correlation coefficients (MCCC, red cross).

3. Spatial comparison of the delay map from rs-fMRI and the TTP map from DSC-MRI

Positive correlations between the sLFO delay time of rs-fMRI and TTP of DSC-MRI were found in all subjects (Fig. S7(a)). The plot was divided into two portions. In the first portion shown in blue (about -4.7 s to -1.7 s), the fitted slope of the line between the TTP from the DSC-MRI and the sLFO delay time from rs-fMRI is roughly flat. Those voxels largely reside in central gyrus and gray matter (as shown in Fig. S7(b)). In the second portion shown in red (about -1.7 s to 2 s), the fitted slope of the line between the TTP and the sLFO delay time is close to 1, which is expected. Those voxels largely reside in some parts of gray matter, white matter, and large draining veins (as shown in Fig. S7(c)). These results demonstrated that the spatial blood flow patterns derived from the rs-fMRI using the sLFO and the DSC-MRI are positively correlated with each other in a large extent. However, we can also see the discrepancy between the delay map from rs-fMRI and the TTP map from the DSC-MRI (See Fig. S8). The potential reasons can be found in the main paper.

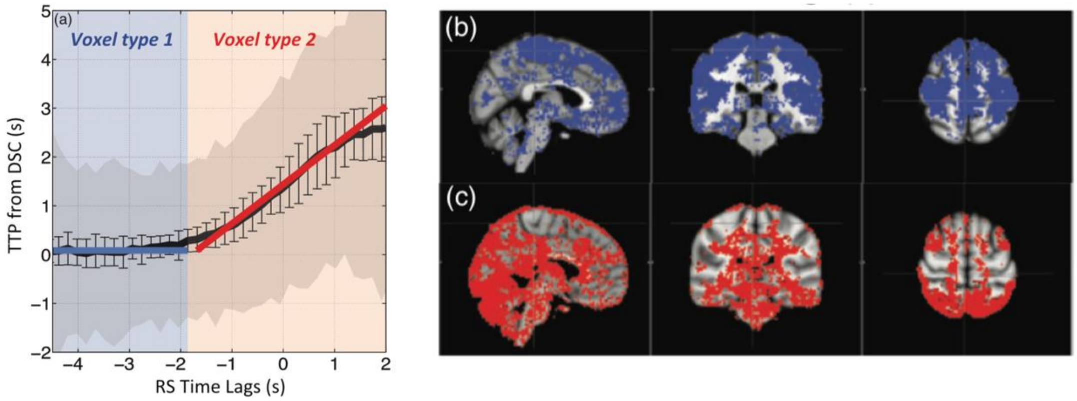

Figure S7: Group results of the TTP from DSC-MRI versus the delay time from rs-fMRI (a). The spatial distribution of voxel type 1 and voxel type 2 are shown in (b) and (c), respectively. This figure was adapted from Tong et al. (2017)

Reference:

1. Tong, Y. et al. Perfusion information extracted from resting state functional magnetic resonance imaging. J Cereb Blood Flow Metab 37, 564-576, doi:10.1177/0271678X16631755 (2017).

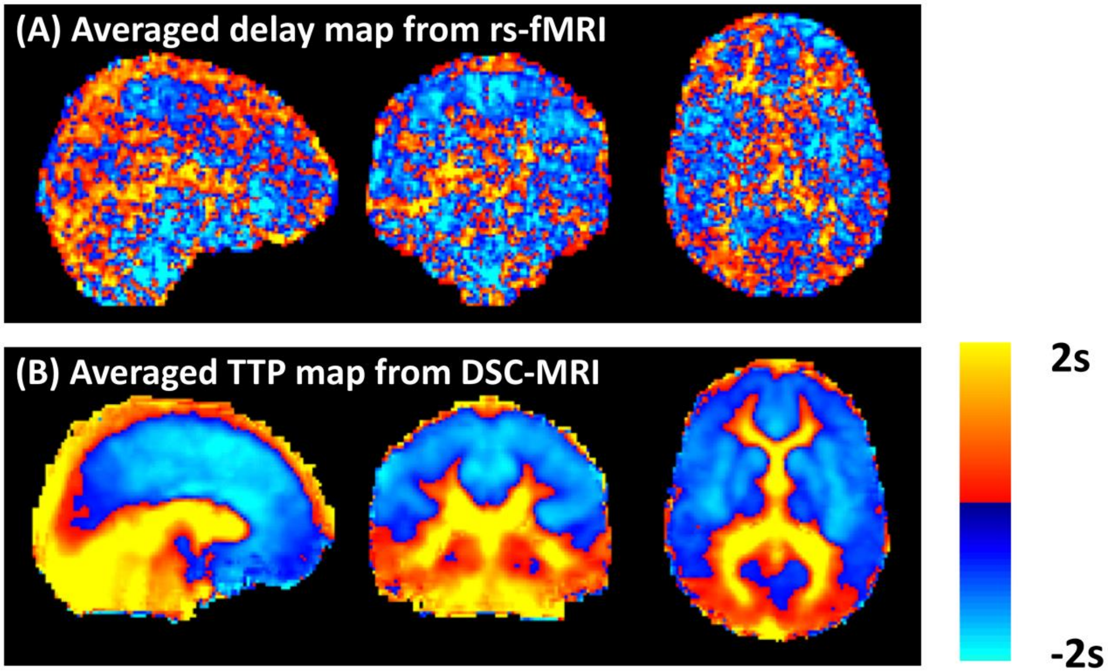

Figure S8: The averaged delay map from rs-fMRI data (A) and the averaged TTP map from DSC-MRI data.

4. Slope-detection algorithm

The slope-detection algorithm supports the choice of two input parameters: *n,* the number of edges to detect, and *edge,* which determines whether edges will be detected on the rising edge (black-to-white; *edge* = -1) or falling edge (white-to-black; *edge* = 1). The chosen carpet plot image (Fig. S3.A) is input as the image matrix *I*. To increase signal to noise ratio (SNR), *I* is first smoothed using the 2D blurring filter

$$h = \frac{1}{144} * \begin{bmatrix} 1 & 3 & 4.25 & 3 & 1 \\ 3 & 8.5 & 12 & 8.5 & 3 \\ 4.25 & 12 & 17 & 12 & 4.25 \\ 3 & 8.5 & 12 & 8.5 & 3 \\ 1 & 3 & 4.25 & 3 & 1 \end{bmatrix}$$

Smoothing via filter *h* is repeated six times with the MATLAB command *filter2*, creating I_{smooth} (Fig. S3.C). I_{smooth} is then averaged along the columns (i.e., over all voxels) to produce an average time series I_{av} (Fig. S3.B). I_{av} is then filtered using the 1D derivative filter h_{d1}, where

$$h_{d1} = \frac{1}{2} * [1 \quad 0 \quad -1] * edge,$$

and the resulting time series is denoted $I_{av,d}$ (Fig. S3.D). The time-locations of the *n* highest peaks of $I_{av,d}$ are selected as the locations at which edge slopes will be computed and are denoted $t_1 \ldots t_n$. If multiple successive peaks occur in the $I_{av,d}$ time series without crossing the zero intensity threshold, only the time-location of the highest peak is recorded.

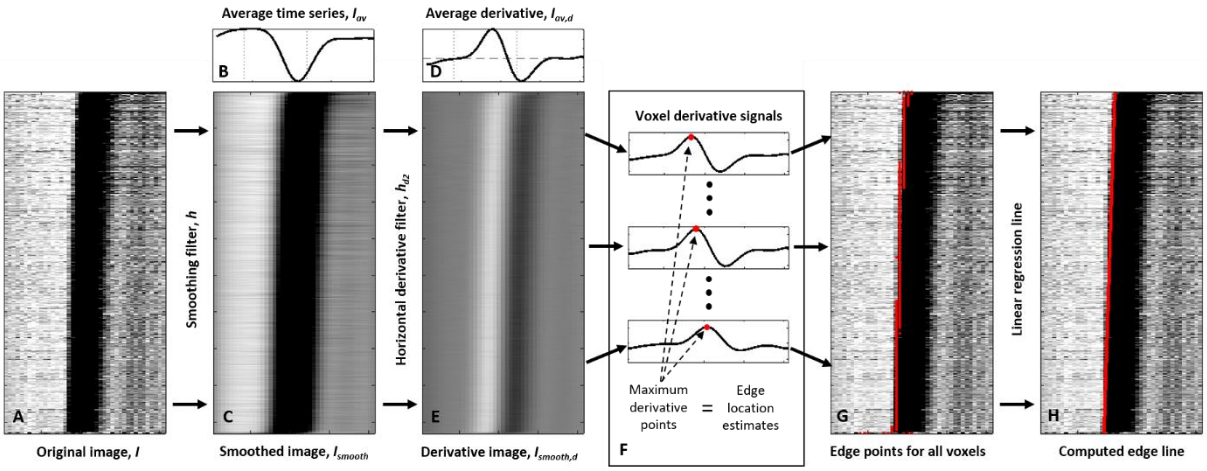

Figure S9. Slope-detecting algorithm developed for computation of carpet plot edge slopes and transit times. A smoothing filter is applied to the original image (A) to produce (C), followed by a horizontal derivative filter to produce (E). (B) and (D) show the average time series after the smoothing and derivative filters, respectively. For every row of (E), the maximum derivative location is located and denoted by a red point as shown in (F). These points are used as data as shown in (G) to compute a linear regression line, shown in (H).

5. Slope and Transit Time Computation

The smoothed carpet plot I_{smooth} is filtered using the 2D horizontal derivative filter h_{d2}, where

$$h_{d2} = \frac{1}{8} * \begin{bmatrix} 1 & 0 & -1 \\ 2 & 0 & -2 \\ 1 & 0 & -1 \end{bmatrix} * edge,$$

producing $I_{smooth,d}$ (Fig. S3.E). For each t_i, the matrix of interest for slope computation is defined by the range of time points immediately surrounding t_i for which $I_{d,av}$ is positive. Two time points are added to each the beginning and end of this range, extending the total range by four time points. The columns of $I_{smooth,d}$ defined by this time range are selected and denoted I_{smooth,d_i}. For each row of I_{smooth,d_i}, the time-location of the maximum row value (which corresponds to the location of highest derivative, i.e., greatest contrast in the given row of I_{smooth}) is recorded (Fig. S3.F). The full set of recorded values forms a set of coordinates (Fig. S3.G) – relating the voxel (row coordinate) to the time point of time location of the edge (column coordinate) – which are used to fit a linear regression line. The slope of this line (Fig. S3.H) represents the estimated slope of the edge present at time location t_i in the original carpet plot I. The estimated time duration traversed by the edge, denoted “transit time” is computed as

$$transit\ time = \frac{n_{voxels}}{slope} * TR.$$

The value n_{voxels} represents the number of voxels included in the (cropped) carpet plot image input into the slope-detection algorithm, *slope* is the computed edge slope, and *TR* is the repetition time for the fMRI scan protocol.

We note that there is some degree of error associated with the computation of the transit times due to time resolution limitations. The computed transit time error can be thought of as a function of three variables: (1) the true underlying transit time, (2) the TR of the imaging sequence, and (3) the time point at which sampling of the true signal begins. The potential error for a given edge is inversely proportional to the true transit time duration, as a longer true duration can be better sampled with a given TR. In the extreme case where the true underlying transit time is less that the TR, then the transit time computation can have an error up to the magnitude of the TR. However, as the true transit time becomes greater than the TR, then the potential error shrinks. In summary, the higher the transit time, the more accurate the measured value. Since we primarily expect transit times in the range of 4-6 seconds, we can expect that the potential error in measurements is much lower than 1.5 seconds.

Tests were performed by creating artificial "perfect" images with a temporal resolution of 0.1 s and tilted edges based on varying true transit times. These artificial plots were then sampled every 15 time points to simulate a TR of 1.5 s, and input into the slope detection algorithm to see how the algorithm performed at measuring the transit time. An example comparison of the computed transit time with the true underlying transit time is shown in Figure S4.A.

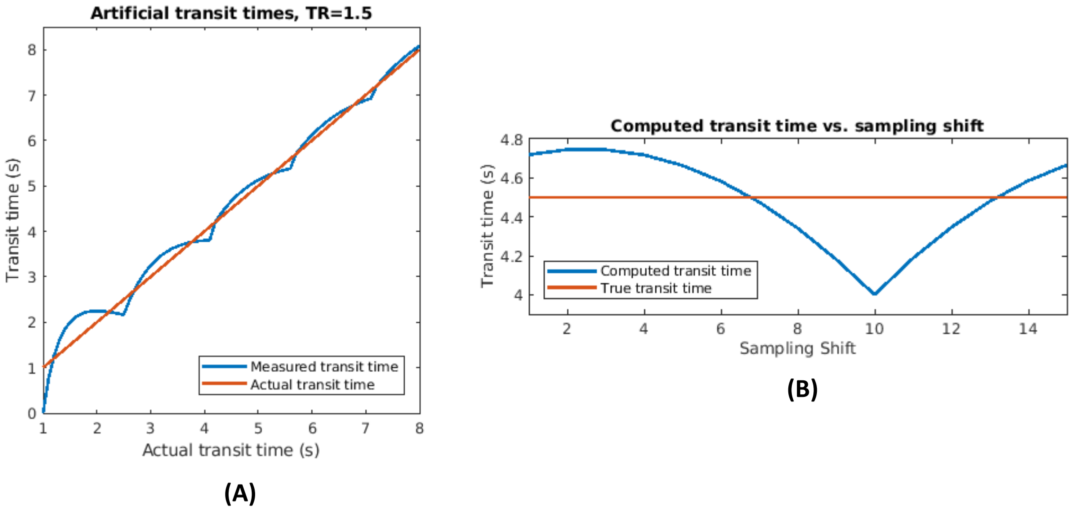

Figure S10. Results of transit time computations on artificial "perfect" edges. Artificial images were created with an edge of a certain true transit time and time resolution of 0.1 s, and then were sampled with a time-resolution of 1.5 s. (A) displays the difference between the computed transit time and the true underlying transit time where the true underlying transit time is varied. (B) displays the difference between the computed transit time and the true underlying transit time when the true transit time is held constant at 4.5 s but the point at which sampling is started is shifted.

As listed as variable (3) above, we also note that the transit time computation will vary based on when this sampling begins within the original image, since (in this case) there are 15 possible “starting points” for the sampling. This represents how the propagation of a sLFO could begin at any point within the given TR. Figure S4.B shows the effect of different sampling starting points on the error of the computed signal for a true transit time of 4.5 s. This shows that we can expect that the error of the transit time computation ranges between approximately -0.6 to 0.3 s for a true underlying time of 4.5 s. It was also observed that this type of error tends to cause the transit time to be underestimated slightly more often than it is overestimated. This is a limitation of the transit time computation method, however we believe that these errors are not significant enough to change the general conclusions we draw in the paper; in particular, these errors are much smaller than the variations in rs-fMRI transit times observed within a single subject.

6. Noise Model Evaluations

The slope-detection algorithm was evaluated for robustness by constructing a simple noise model to analyze how consistently the algorithm performed under variation of two parameters: intensity contrast and noise level. Within this model, carpet plot images were assumed to consist of two components: an underlying clean image and an added image of Gaussian noise. The contrast value for a given carpet plot was estimated by computing the difference between the average value over selected regions of highest values (lightest) and that of selected regions of lowest value (darkest). The noise level of a carpet plot was estimated as follows: the underlying clean image was estimated by applying a 2D smoothing filter (identical to that of the slope-detecting algorithm) to the original image. The noise image was then estimated by subtracting the clean image estimate from the original. The standard deviation over all values in the estimated noise image was then computed and formed the quantified noise level of the given carpet plot. Rs-fMRI carpet plots produced contrasts in the range 0.735-1.471 and noise standard deviations in the range 0.737-0.817. DSC-MRI carpet plots produced contrasts in the range 2.899-3.226 and noise standard deviations in the range 0.197-0.330. Due to the processing and scaling of the fMRI data these measurements are simply in units of signal intensity of the processed carpet plot data.

This model was developed in order to evaluate the reliability of the slope-detection algorithm, completed by scaling a chosen DSC carpet plot to varying contrast (ranging from 0.5 - 3.5) and noise levels (ranging from 0.1 - 1.0) and conducting repeated trials (n = 30) of random noise addition at each pairing of contrast and noise level to determine the consistency of transit time computation. Scaling of the chosen DSC carpet plot to varying contrast and noise levels was completed as follows: a single cropped DSC carpet plot was chosen to serve as the baseline image and was smoothed using the filter h described above. Then, for a given trial, the smoothed image was scaled to a specified contrast level (completed by scaling the image by a factor defined by the ratio between the desired contrast level and the contrast level of the selected DSC carpet plot) and was added to an equally-sized image of random Gaussian noise at a specified standard deviation. 30 trials of each combination of contrast (ranging from 0.5-3.5) and random noise at a

given standard deviation (ranging from 0.1-1.0) were completed. The results of this analysis are given in Figure 7 in the main document.

We note that this noise model represents an oversimplification of the noise observed in rs-fMRI data. The noise which corrupts the BOLD signal is not simply Gaussian in nature, and it may not be ultimately correct to assume perfect and linear underlying edges exist beneath the noise in carpet plot images. For instance, Figure S11 displays a DSC carpet plot image which has been scaled to the contrast and noise level of RS carpet plots compared to an unaltered RS carpet plot image. We note that despite being “equal” in contrast and noise level to the RS image, the DSC image still appears to contain a bolder and cleaner edge that the RS image, indicating that our noise model does not fully capture the characteristics which impact the quality of edges seen in carpet plots. These observations limit our ability to yet claim causal physiological differences between edges of different transit time.

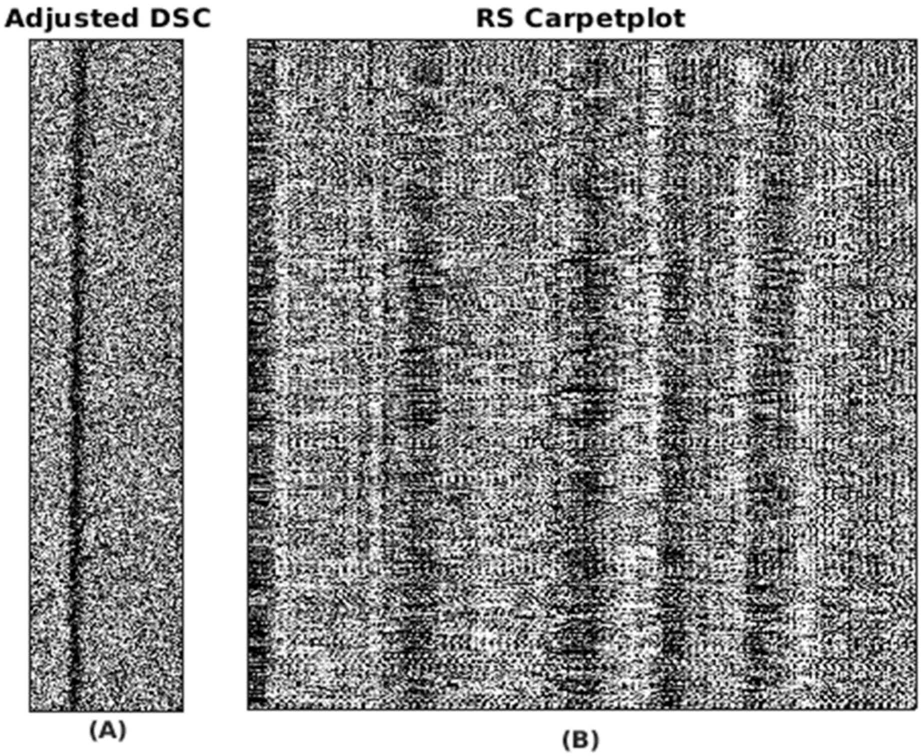

Figure S11. Adjusted DSC carpet plot compared to RS carpet plot. In (A), a DSC carpet plot is scaled to the contrast and noise level of a RS carpet plot. (B) displays an unaltered RS carpet plot. These figures illustrate that under this noise model, the adjusted DSC carpet plot still displays a stronger line than the actual RS data.

Given these limitations, an additional test was run to evaluate the consistency of slope detection where the noise model was used to “reconstruct” the input carpet plots. To complete this “reconstruction”, a given carpet plot was first smoothed using the filter *h* described above. Then an image of Gaussian noise at standard deviation defined by that carpet plot’s noise level

(computed as described previously) was added back into the smoothed image. The goal of these operations was to recreate a given carpet plot with an equivalent contrast and random noise level so that the resulting transit time computations could be analyzed. 30 trials were completed for each DSC and RS carpet plot, where this reconstruction process was applied and the slope-detection algorithm was then used to find a single edge's transit time (that of the most prominent edge as detected by the algorithm). Results are displayed in Figure S12. We note that application of the reconstruction process resulted in a slight increase in transit time for some carpet plots, while it resulted in a slight decrease in transit time for others, indicating no obviously clear pattern. The reconstruction trials resulted in a wider variance of transit times for RS carpet plots compared to those from DSC imaging, which is to be expected due to higher SNR in DSC data. While the size of this variance varied slightly from subject to subject, the resulting standard deviations were all small relative to the variance in transit times observed across different time points for a given subject, indicating that the variance in transit time for different edges within the same carpet plot was likely due to true underlying differences as opposed to algorithm error.

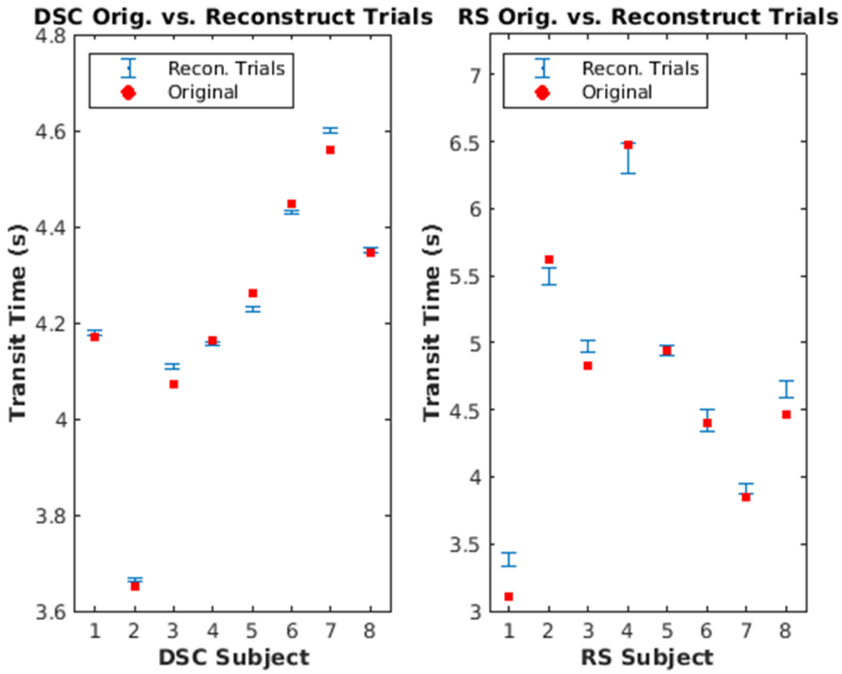

Figure S12. Noise model reconstruction of DSC and RS carpet plots. For each subject's DSC and RS dataset, the carpet plot was filtered to remove noise, and the standard deviation of removed noise was computed. Random white noise at the computed standard deviation was then added back to the filtered image (creating the reconstructed image) and the transit time recomputed. This was completed for 30 trials per carpet plot. Red points indicate the original transit time for the first slope detected in the unaltered carpet plot. Vertical bars for reconstructed trials (blue) are twice as long as the standard deviation of resulting transit times and centered at the mean for that subject.
